# The transcription factor IRF4 regulates the homeostasis and function of intestinal ILC3s

**DOI:** 10.1016/j.isci.2025.112800

**Published:** 2025-05-31

**Authors:** Xianzhi Gao, Xin Shen, Qianying Xu, Yan Zeng, Linjia Dong, Shenghui Hong, Huihui Jin, Qianqian Wang, Di Wang, Linrong Lu, Lie Wang

**Affiliations:** 1 Liang Zhu Laboratory, Zhejiang University Medical Center, Hangzhou, China; 2 Institute of Immunology and Bone Marrow Transplantation Center, First Affiliated Hospital, Zhejiang University School of Medicine, Hangzhou, China; 3 Co-facility centre, Zhejiang University School of Medicine, Hangzhou, Zhejiang 310058, P.R. China; 4 Laboratory Animal Center, Zhejiang University, Hangzhou, China; 5 School of Basic Medical Sciences and Forensic Medicine, Hangzhou Medical College, Hangzhou, Zhejiang, China; 6 Zhejiang University School of Medicine, Hangzhou, China

**Keywords:** Health sciences, Biological sciences, Molecular biology, Molecular biology experimental approach

## Abstract

Group 3 innate lymphoid cells (ILC3s) serve as critical guardians of mucosal immunity. However, the transcriptional networks governing their function remain incompletely characterized. Here, we demonstrate that interferon regulatory factor 4 (IRF4) is essential for maintaining intestinal ILC3 homeostasis and function. IRF4-deficient mice exhibit reduced NKp46^+^ ILC3s, expanded precursor-like NKp46^−^CCR6^−^ ILC3s, and impaired interleukin-22 (IL-22)/IL-17A production, increasing susceptibility to infections. Furthermore, IRF4 loss disrupted major histocompatibility complex (MHC)-class II-associated transcriptional signatures in ILC3s, particularly in CCR6^+^ ILC3s, accompanied by downregulation of MHC class II protein expression. This perturbation consequently diminished ILC-mediated apoptosis of effector CD4^+^ T cells. Sequencing and trajectory analysis link IRF4 to NKp46^+^ ILC3 maintenance and Tbx21 regulation. ATAC-seq/CUT&Tag reveal direct IRF4 binding to *Batf*, *Tbx21*, *Il22*, *Il17a*, and MHC II loci. Overexpression of T-bet partially rescued the differentiation defects in intestinal ILC3s, whereas Batf overexpression partially restored functional impairments and significantly enhanced MHC class II expression in ILC3s.

## Highlights

• IRF4 is a master regulator of intestinal NKp46⁺ ILC3 homeostasis and function
• Revealing IRF4’s dual role in sustaining MHC-II⁺ ILC3s-mediated T-cell tolerance via BATF
• Integrating multi-omics to map IRF4’s direct binding to *Batf*, *Tbx21*, *Il22*, and MHC-II loci
• Demonstrating functional rescue of IRF4-deficient ILC3s by T-bet/BATF overexpression

## Graphical abstract

**Figure:**
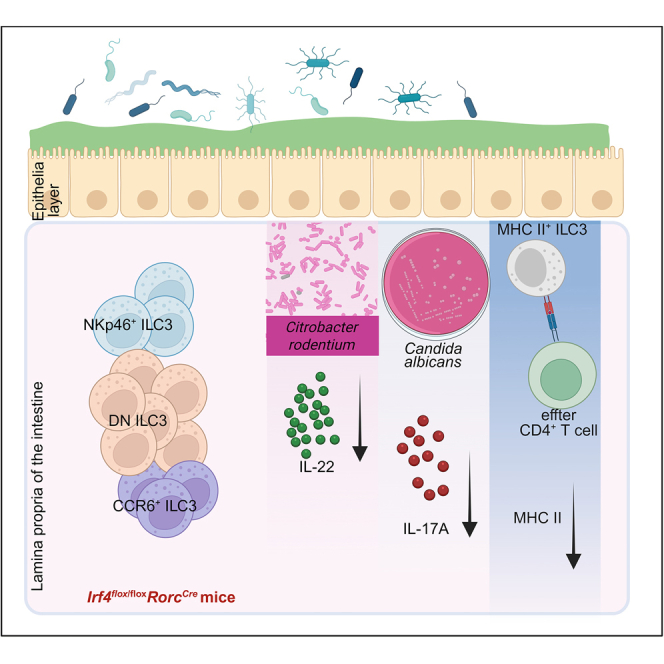

## Introduction

Group 3 innate lymphoid cells (ILC3s) are predominantly localized at tissue surfaces and play a critical role in host defense against infections. Based on the expression of natural cytotoxicity receptor NKp46 (NCR1) and chemokine receptor CCR6, adult ILC3s can be categorized into three subsets: NKp46^+^ ILC3s, CCR6^+^ ILC3s, and NKp46^−^CCR6^−^ ILC3s.^1^^,^^2^ Upon activation, ILC3s secrete a range of cytokines essential for maintaining mucosal barrier integrity. Notably, RORγt-expressing ILC3s serve as key innate producers of interleukin-17A (IL-17A) and IL-22, which are crucial for modulating immune responses against extracellular bacteria and autoimmune diseases.^3^^,^^4^^,^^5^ Among these subsets, CCR6^+^ ILC3s have been shown to express major histocompatibility complex (MHC) class II molecules, enabling them to process and present antigens to CD4^+^ T cells.^6^^,^^7^^,^^8^ However, intestinal MHC class II^+^ ILC3s exhibit minimal expression of classical co-stimulatory molecules such as CD80 and CD86.^7^^,^^9^ Intriguingly, MHC class II + ILC3s have been demonstrated to suppress microbial-specific CD4^+^ T cell responses and mitigate intestinal inflammation.^10^

Multiple transcription factors known to regulate T cell development also play pivotal roles in ILC3 biology, including RORγt,^11^ T-bet,^12^ and GATA3.^13^^,^^14^ Among these, RORγt is indispensable for ILC3 development and core functions such as IL-22 production.^15^ GATA3 modulates ILC3 homeostasis by regulating IL-7Rα expression and mediating the interplay between RORγt and T-bet.^16^^,^^17^ NKp46^−^CCR6^−^ ILC3s represent a plastic precursor population capable of differentiating into NKp46^+^ ILC3s under the influence of a T-bet expression gradient and its downstream Notch signaling pathway.^18^^,^^19^ Beyond these factors, ThPOK (T-helper-inducing POZ/Krüppel-like factor) and RUNX3 serve as central regulators of ILC3 lineage commitment. Our previous work demonstrated that ThPOK-deficient mice exhibit selective depletion of NKp46^+^ ILC3s alongside an expansion of NKp46^−^CCR6^−^ ILC3s, resulting in impaired resistance to *Salmonella typhimurium* infection.^20^ RUNX3 further contributes to ILC3 differentiation by binding the *Rorc* promoter to drive expression of RORγt and AHR, both essential for intestinal ILC1 and ILC3 development.^21^^,^^22^

Interferon regulatory factor 4 (IRF4) serves as a master regulator of differentiation, homeostasis, and function across multiple T cell subsets, including Th1, Th2, Th17, and T follicular helper (Tfh) cells. Studies demonstrate that IRF4 sustains Th1 cell development, as IRF4-deficient CD4^+^ T cells exhibit impaired Th1-associated gene expression upon *in vitro* stimulation.^23^ Furthermore, IRF4 is specifically required for GATA3-mediated Th2 differentiation, IL-4 production regulation, and Th2 cell suppression by regulatory T cells.^24^^,^^25^ In Th17 cell biology, IRF4 plays an essential role in development and mediates protective responses against intestinal pathogens.^26^ Additionally, IRF4 represents an intrinsic requirement for Tfh cell differentiation and germinal center formation.^27^ Beyond adaptive immunity, IRF4 contributes to innate immune responses by regulating ILC2-mediated lung epithelial reactivity^28^ and orchestrating natural killer (NK) cell activation and differentiation.^29^

Given the critical role of IRF4 in CD4^+^ T cells and the shared cytokine profile and transcriptional regulators between T cells and ILC3s, we sought to elucidate IRF4’s function in ILC3 biology. Our findings demonstrate that IRF4 intrinsically maintains intestinal NKp46^+^ ILC3 homeostasis. IRF4 deficiency resulted in impaired IL-22 and IL-17A production by ILC3s and increased host susceptibility to *Citrobacter rodentium* and *Candida albicans* infections. Single-cell RNA sequencing (scRNA-seq) analysis revealed that IRF4 sustains NKp46^+^ ILC3 homeostasis, with IRF4 deficiency causing significant transcriptional alterations, including downregulation of *Tbx21*, *Runx3*, and *Gata3*. Integrated analysis of ATAC-seq and CUT&Tag datasets identified direct IRF4 binding to *Batf*, *Tbx21*, *Il22*, and *Il17a* loci. Notably, T-bet overexpression rescued the differentiation defects observed in both NKp46^+^ and double-negative (DN) ILC3 populations. Consistent with our scRNA-seq data, IRF4 deficiency reduced MHC class II expression in CCR6^+^ ILC3s, leading to diminished ILC3-mediated apoptosis of effector CD4^+^ T cells. IRF4 directly bound to multiple MHC class II-associated loci (*H2-DMa*, *H2-Aa*, *H2-D1*, *H2-Eb1*, and *H2-Oa*), establishing its direct role in regulating MHC class II gene expression. Notably, while mock-transfected common lymphoid progenitors (CLPs) generated IRF4-deficient ILC3s with minimal IL-22 and IL-17A production, *Batf* transfection restored robust cytokine production and significantly enhanced MHC class II expression in ILC3s.

## Results

### IRF4 deletion in ILC3s leads to homeostatic dysregulation

We systematically analyzed IRF4 expression across innate lymphoid cell subsets, including ILC1, ILC2, and ILC3 populations (NKp46^+^ ILC3s, CCR6^+^ ILC3s, and NKp46^−^CCR6^−^ ILC3s). Quantitative analysis revealed constitutive IRF4 expression in all three ILC lineages, with significantly higher levels observed in ILC3s compared to other subsets (Figure S1A). Flow cytometric assessment demonstrated comparable IRF4 expression between NKp46^+^ ILC3s and NKp46^−^CCR6^−^ ILC3s based on mean fluorescence intensity (Figure S1B). Following *C. rodentium* infection, we observed upregulated IRF4 expression in all ILC3 subsets (NKp46^+^, CCR6^+^, and NKp46^−^CCR6^−^ ILC3s) (Figures S1C and S1D). *In vitro* stimulation with IL-23 and IL-1β also significantly enhanced IRF4 expression in ILC3s (Figure S1E). These findings collectively suggest a potentially important functional role for IRF4 in ILC3 biology.

**Figure 1:**
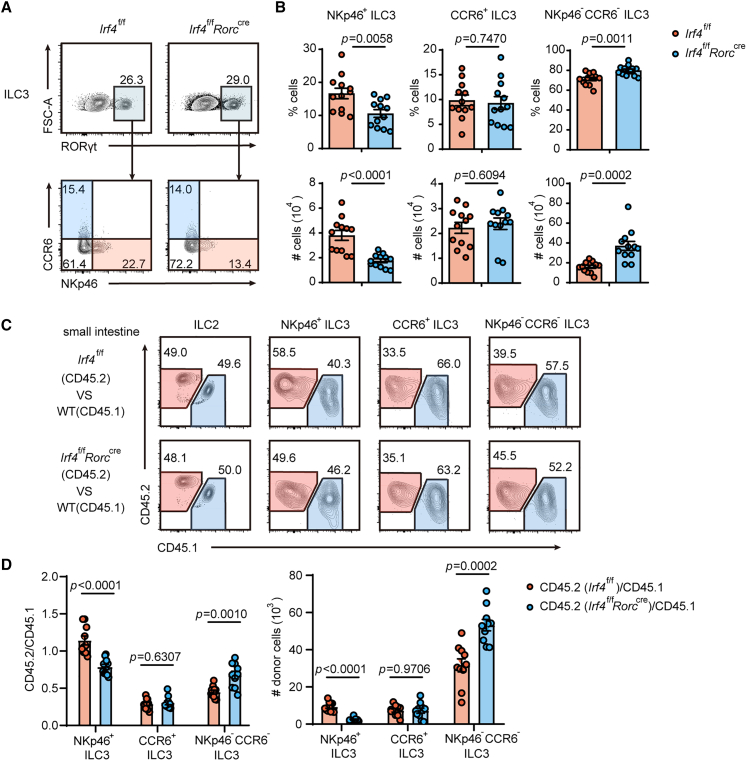
IRF4 cell-intrinsically regulated ILC3 homeostasis (A) Flow cytometry of ILC3 subsets isolated from the siLP of *Irf4*^f/f^ and *Irf4*^f/f^*Rorc*^cre^ mice. (B) The percentages and total cell numbers of three subsets were compared (*n* = 12). (C and D) Bone marrow chimeras. (C) The expression of CD45.1 and CD45.2 in ILC2s and ILC3 subsets isolated from the siLP of reconstituted mice. (D) The percentages and cell numbers of donor-derived cells shown in (C) (CD45.2/CD45.1) were compared (*n* = 10). Bar graphs are presented as mean ± SEM. A two-tailed Student’s t test was performed for comparisons. The data are representative of at least three independent experiments (A–D). ∗∗*p* < 0.01, ∗∗∗*p* < 0.001. See also Figures S1 and S2.

We crossed *Irf4*^f/f^ mice with *Rorc*-cre mice and confirmed successful IRF4 ablation within intestinal ILC3 subsets of these conditional knockout mice (Figure S1F). Analysis revealed that although the overall proportion of RORγt^+^ ILC3s remained unchanged in IRF4-deficient mice (Figure 1A), we observed significant alterations in ILC3 subset distribution. Notably, NKp46^+^ ILC3s exhibited a selective reduction in both absolute cell numbers and frequencies (Figure 1B). Conversely, the NKp46^−^CCR6^−^ ILC3 population was significantly expanded within the Lin-RORγt^+^ ILC3 compartment, whereas CCR6^+^ ILC3s and CCR6^+^CD4^+^ ILC3s remained roughly unaffected (Figures 1B, S1K, and S1L). Importantly, other ILC subsets (ILC1s and ILC2s) showed no significant changes in IRF4-deficient mice (Figures S1G–S1J). To determine the mechanism underlying these phenotypic changes, we assessed cellular proliferation and survival. Neither apoptosis (Annexin V^+^ cells) nor proliferation (Ki67^+^ cells) rates differed significantly among ILC3 subsets between knockout and control mice (Figures S2A–S2D), excluding these processes as contributing factors. Furthermore, IRF4 deficiency did not impair early ILC development, as evidenced by comparable numbers and frequencies of ILC progenitors that give rise to the adult ILC pool (Figures S2E–S2H).

Given the established crosstalk between ILCs and T helper cells,^30^ we sought to determine whether the homeostasis of IRF4-deficient intestinal ILC3 subsets was regulated through cell-intrinsic mechanisms. To address this, we generated competitive bone marrow chimeras where IRF4-deficient and control cells repopulated in equal competition. Specifically, we mixed CD45.2+ bone marrow cells from either *Irf4*^f/f^ or *Irf4*^f/f^ *Rorc*^cre^ mice with CD45.1^+^ wild-type bone marrow cells at a 1:1 ratio, then intravenously transplanted these into sublethally irradiated CD45.1^+^ recipient mice. Eight weeks post-transplantation, we analyzed gut lamina propria lymphocytes (LPLs) derived from CD45.2^+^ donor cells by flow cytometry. Notably, both *Irf4*^f/f^ and *Irf4*^f/f^ *Rorc*^cre^ bone marrow cells successfully reconstituted intestinal ILC2 populations at comparable frequencies (Figure 1C), suggesting that IRF4 deficiency does not intrinsically impair ILC2 development or maintenance. This competitive repopulation assay allowed us to distinguish cell-intrinsic effects from potential microenvironmental influences on ILC3 subset homeostasis.

Consistent with the phenotype observed in IRF4-deficient mice, competitive bone marrow chimeras receiving IRF4-deficient cells exhibited a specific reduction in NKp46^+^ ILC3 numbers (Figures 1C and 1D), demonstrating that IRF4 intrinsically regulates NKp46^+^ ILC3 maintenance. Furthermore, we detected a significant expansion of NKp46^−^CCR6^−^ ILC3s derived from IRF4-deficient donor cells compared to their wild-type counterparts. In contrast, CCR6^+^ ILC3 frequencies remained comparable between genotypes. These findings establish that IRF4 cell-intrinsically governs the homeostasis of ILC3 subsets in the intestinal compartment.

### IRF4 mediates IL-22 and IL-17A secretion in ILC3s and alters the susceptibility to infection

**Figure 2:**
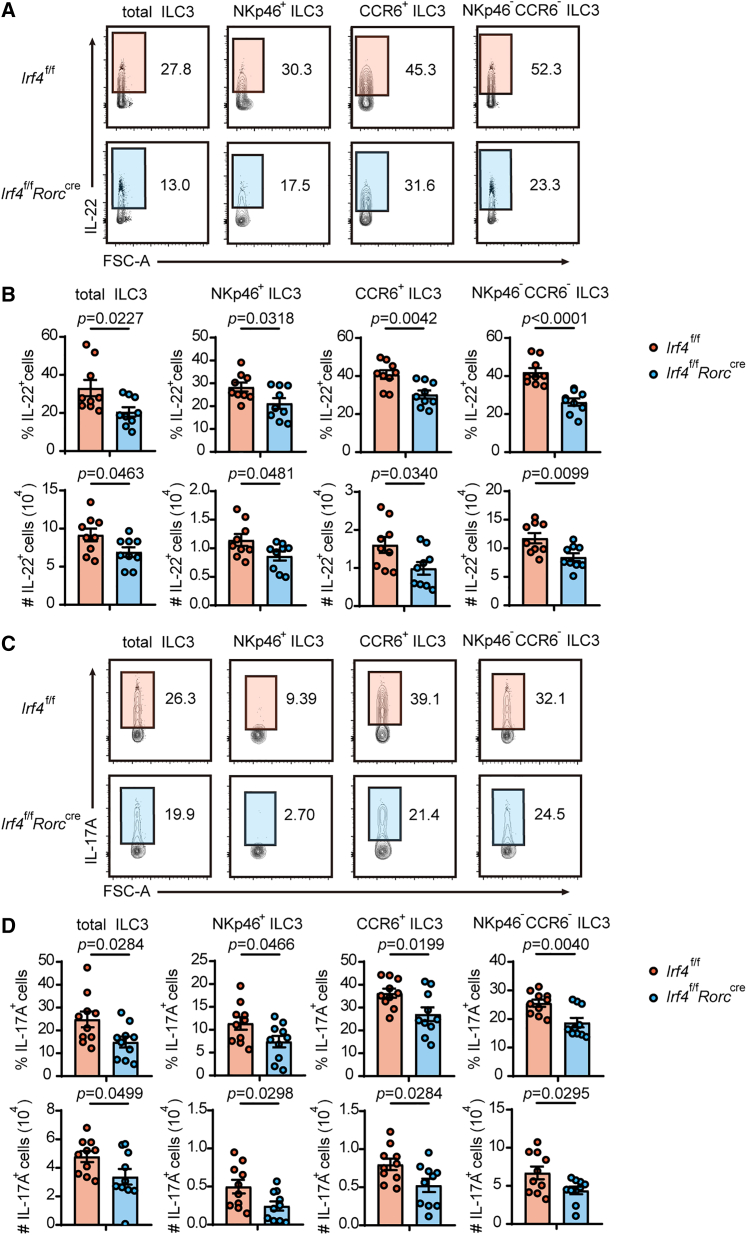
IRF4-deficient ILC3s display impaired cytokine production under stimulation *in vitro* (A–D) Cytokine production in siLP ILC3s from *Irf4*^f/f^ and *Irf4*^f/f^*Rorc*^cre^ mice. (A and B) Representative flow plots (A) and quantification (B) of IL-22 production from total ILC3s, NKp46^+^ ILC3s, CCR6^+^ ILC3s, and NKp46^−^CCR6^−^ ILC3s after stimulation with IL-1β and IL-23 (*n* = 9). (C and D) Representative flow plots (C) and quantification (D) of IL-17A production from total ILC3s, NKp46^+^ ILC3s, CCR6^+^ ILC3s, and NKp46^−^CCR6^−^ ILC3s after stimulation with PMA and Ionomycin (*n* = 10). Bar graphs are presented as mean ± SEM. A two-tailed Student’s t test was performed for comparisons. The data are representative of at least three independent experiments (A–D). ∗*p* < 0.05, ∗∗*p* < 0.01, ∗∗∗*p* < 0.001.

To investigate whether the reduced NKp46^+^ ILC3 population in IRF4-deficient mice correlated with functional impairments, we examined cytokine production in stimulated ILC3 subsets. IL-22-producing cells were approximately 50% less abundant in IRF4-knockout mice compared to wild-type controls, especially in NKp46^+^ ILC3s and NKp46^−^CCR6^−^ ILC3s, with the latter showing a more profound reduction (Figures 2A and 2B). Similarly, IRF4 deficiency significantly compromised IL-17A production in total ILC3s (Figures 2C and 2D). Subset analysis revealed that both CCR6^+^ ILC3s and NKp46^−^CCR6^−^ ILC3s exhibited markedly diminished IL-17A secretion. These findings demonstrate that IRF4 is essential for maintaining the functional competence of intestinal ILC3 subsets, particularly their capacity to produce key effector cytokines.

**Figure 3:**
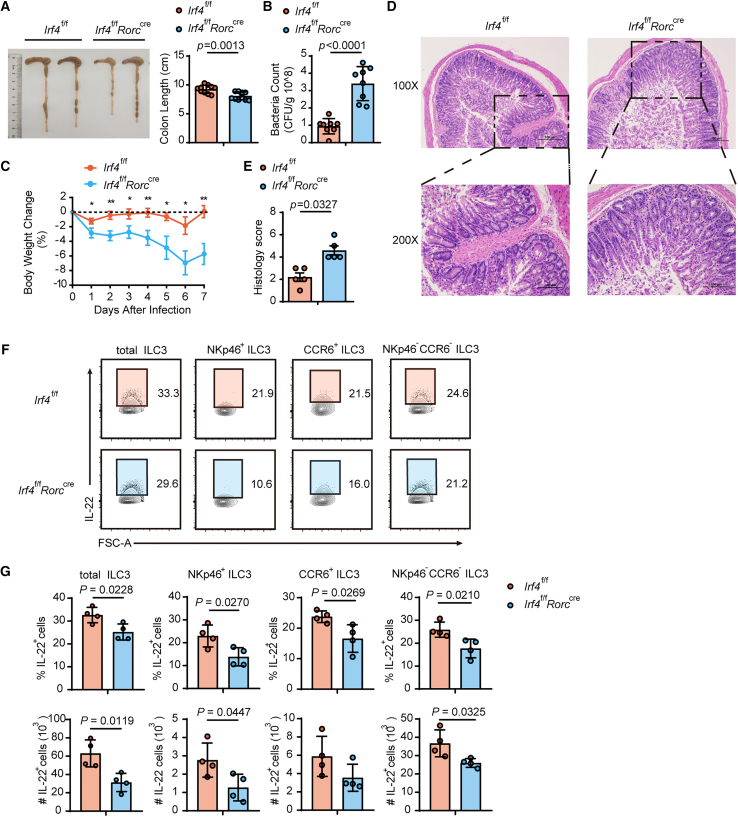
The defective functionality of ILC3s in IRF4-deficient mice compromises the host’s defense against *C. rodentium* infection (A–H) *C. rodentium* infection model. (A) Colon lengths of *Irf4*^f/f^ and *Irf4*^f/f^*Rorc*^cre^ mice were measured and compared in a bar graph. (*n* = 10). (B) *C. rodentium* CFUs in feces 7 days after infection (*n* = 8). (C) Body weight changes of *Irf4*^f/f^ and *Irf4*^f/f^*Rorc*^cre^ mice after infection at the indicated time points. Mice were excluded from analysis after death (*n* = 10). (D) Histological analysis of colonic tissues by H&E staining. (E) Pathological score of colon histology. (F and G) The percentages and numbers of IL-22^+^ ILC3 cells in the large intestine were compared (*n* = 4). Bar graphs are presented as mean ± SEM. A two-tailed Student’s t test was performed for comparisons. The data are representative of at least three independent experiments (A–H). ∗*p* < 0.05, ∗∗*p* < 0.01, ∗∗∗*p* < 0.001.

Given the crucial role of ILC3-derived cytokines in infection-induced colitis, particularly IL-22-mediated clearance of *Citrobacter rodentium*,^31^ we evaluated the consequences of IRF4 deficiency during bacterial challenge. *Irf4*^f/f^ *Rorc*^cre^ mice infected with *C. rodentium* displayed significant pathological manifestations, including pronounced colon shortening, elevated fecal bacterial burdens, and accelerated weight loss compared to wild-type controls (Figures 3A–3C). Histopathological analysis revealed more severe intestinal damage in knockout mice, characterized by crypt hyperplasia, luminal exudates, and extensive immune cell infiltration (Figures 3D and 3E). Mechanistically, these exacerbated disease outcomes correlated with impaired IL-22 production, as *Irf4*^f/f^ *Rorc*^cre^ mice exhibited significantly fewer IL-22-producing ILC3s in the large intestine (Figures 3F and 3G). This defect was consistent across all ILC3 subsets (NKp46^+^, CCR6^+^, and NKp46^−^CCR6^−^), demonstrating that IRF4 is essential for maintaining protective cytokine responses during enteric bacterial infection.

**Figure 4:**
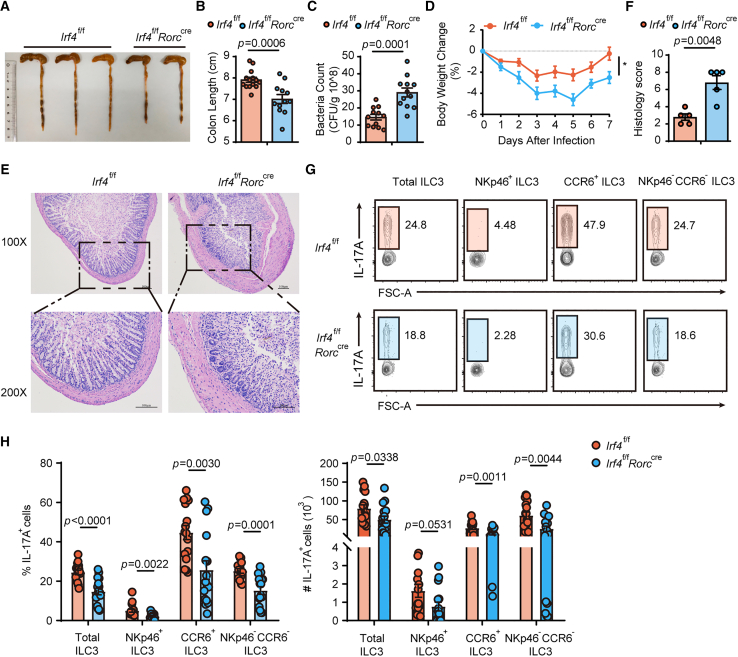
*Irf4*^f/f^*Rorc*^cre^ mice are more sensitive to *C. albicans* infection with decreased IL-17A production (A–H) *C. albicans* infection model. (A) Representative image of colons in *Irf4*^f/f^ mice and *Irf4*^f/f^*Rorc*^cre^ mice after *C. albicans* infection on day 7. (B) Colon lengths were counted and plotted in infected mice (*n* = 14 WT, *n* = 12 KO). (C) Colonies of *C. albicans* in the feces were counted by serial dilution (*n* = 12). (D) Body weight changes (*n* = 12). (E) H&E-stained sections of representative colons. (F) Pathological score of colon histology. (G) Flow cytometric analysis of IL-17A expression in the small intestine 7 days after infection. (H) The numbers and percentages of IL-17A^+^ cells were compared (*n* = 16). Bar graphs are presented as mean ± SEM. A two-tailed Student’s t test was performed for comparisons. The data are representative of at least three independent experiments (A–H). ∗*p* < 0.05, ∗∗*p* < 0.01, ∗∗∗*p* < 0.001.

To further characterize the functional consequences of impaired IL-17A production in IRF4-deficient ILC3s, we employed a murine oropharyngeal *C. albicans* infection model, where IL-17-secreting ILC3s are known to mediate antifungal immunity.^32^ IRF4-deficient mice exhibited exacerbated disease progression following *C. albicans* challenge, as evidenced by shorter colon lengths, elevated fungal loads in fecal samples, progressive weight loss, and increased histopathological scores reflecting severe intestinal inflammation (Figures 4A–4F). Consistent with the observed pathology, flow cytometric analysis revealed markedly reduced IL-17A production across all small intestinal ILC3 subsets in IRF4-deficient mice compared to wild-type controls (Figures 4G and 4H). These results establish IRF4 as a critical regulator of ILC3-mediated antifungal responses through its control of IL-17A production.

### The effect of IRF4 on ILC3s functionality is cell intrinsic

**Figure 5:**
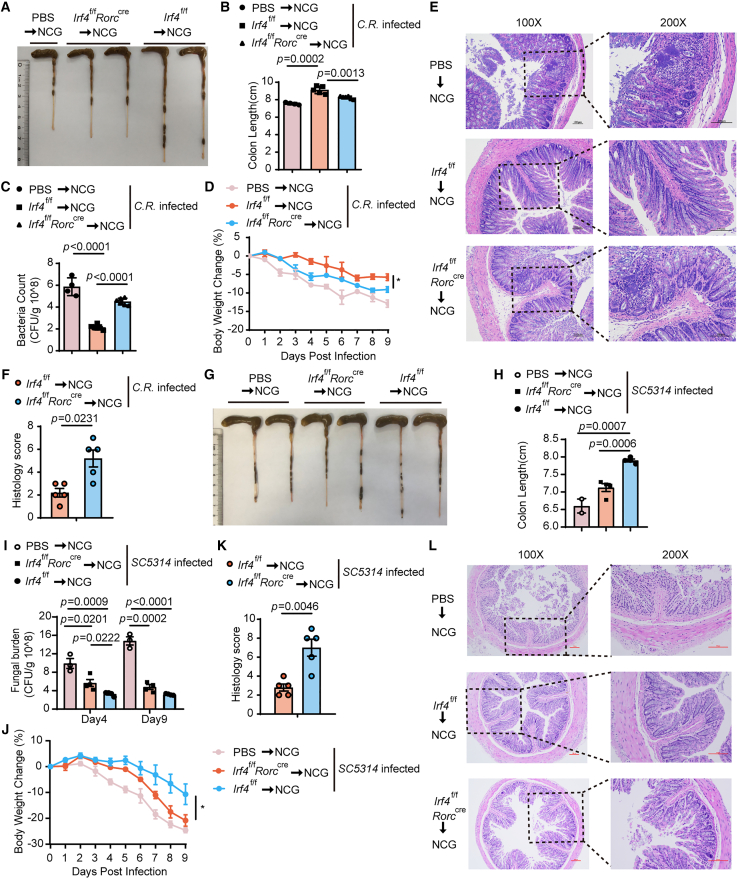
Defective ILC3 function in IRF4-deficient mice drives *C. rodentium* and *C. albicans* infection in a cell-intrinsic manner (A–F) NCG mice were adoptively transferred with 80,000 intestinal ILC3s (Lin^−^CD127^+^CD27^−^KLRG1^−^) sorted from the small intestine of *Irf4*^f/f^ and *Irf4*^f/f^*Rorc*^cre^ mice or PBS as control after being treated with ABX for 1 week. ILC3s were stimulated with IL-23 and IL-1β for 30 min before injected into NCG mice through the tail vein. NCG mice were orally inoculated with *C. rodentium* 24 h after adoptive transfer. (A, B) Measurements (A) and statistical analysis (B) of the colon lengths from NCG recipients (*n* = 4 control, *n* = 5 WT transferred, *n* = 7 KO transferred). (C) CFUs in the feces of NCG recipients 9 days after infection (*n* = 4 control, *n* = 5 WT transferred, *n* = 7 KO transferred). (D) Changes in body weight were recorded at the indicated time points (*n* = 4 control, *n* = 5 WT transferred, *n* = 7 KO transferred). (E) H&E staining of colon tissue sections. (F) Pathological score of colon histology. (G–L) *Irf4*^f/f^ or *Irf4*^f/f^*Rorc*^cre^ ILC3s were adoptively transferred into NCG mice with *C. albicans* infection. (G and H) Measurements (G) and statistical analysis (H) of the colon lengths from NCG recipients (*n* = 2 control, *n* = 4 transferred). (I) Fecal fungal burden (*n* = 3 control, *n* = 4 transferred). (J) Variations of body weight were shown each day (*n* = 4). (K) Pathological score of colon histology. (L) Histological analysis of colonic tissues by H&E staining. The data are representative of at least two independent experiments (A–L). Bar graphs are presented as mean ± SEM. A two-tailed Student’s t test was performed for comparisons. ∗*p* < 0.05, ∗∗*p* < 0.01, ∗∗∗*p* < 0.001. See also Figures S3.

Efficient control of pathogenic microbial infection in mice requires synergy between the innate and adaptive immune systems.^33^ Both Th17 and Tregs play an essential role in the infection experiments and are also affected by the conditional deletion of IRF4. To rule out the effects of the acquired immune system *in vivo*, we performed adoptive transfer of intestinal ILC3s purified from either *Irf4*^f/f^ *Rorc*^cre^ or *Irf4*^f/f^ mice into NCG mice, where neither ILCs nor T/B cells are produced, to determine whether ILC3s of IRF4-competent mice alone can protect NCG mice. The NCG reconstitution assay has been extensively utilized in ILC3 research, with our experimental data confirming efficient engraftment of adoptively transferred ILC3s in the intestinal mucosa of NCG mice (Figures S3A and S3B). Nine days later, the colon length of the mice injected with IRF4-deficient ILC3 cells was markedly shorter than those receiving wild-type (WT) ILC3 cells (Figures 5A and 5B). The colony-forming units (CFUs) of *C. rodentium* in the feces collected from NCG mice injected with deficient ILC3s were approximately 2-fold higher than that from NCG mice receiving WT ILC3s (Figure 5C). Collectively, ILC3s derived from deficient mice lost their protective capability, especially against weight loss and intestinal injury, compared to WT intestinal ILC3s (Figures 5D–5F).

We also sorted intestinal ILC3s from *Irf4*^f/f^ or *Irf4*^f/f^ *Rorc*^cre^ mice and adoptively transferred target cells into NCG immunodeficient mice and then generated a *C. albicans* infection model. Nine days after infection, the NCG mice receiving IRF4-deficient ILC3s developed more severe illness than those receiving *Irf4*^f/f^-ILC3 cells. Our pathological findings were in line with the *C. albicans* infection model, implying insufficient clearance of *C. albicans*. Specifically, the colon length of the NCG mice receiving IRF4-deficient cells was shorter (Figures 5G and 5H), the *C. albicans* fungal burden was higher (Figure 5I), their body weight decreased faster (Figure 5J), and the damage to the colon epithelial barrier was more pronounced (Figures 5K and 5L). In conclusion, the exacerbation of pathogenic microbial infection in IRF4-deficient mice was mediated in a cell-intrinsic manner.

### Single-cell RNA sequencing indicates that IRF4 specifically preserves the NKp46^+^ ILC3 identity

**Figure 6:**
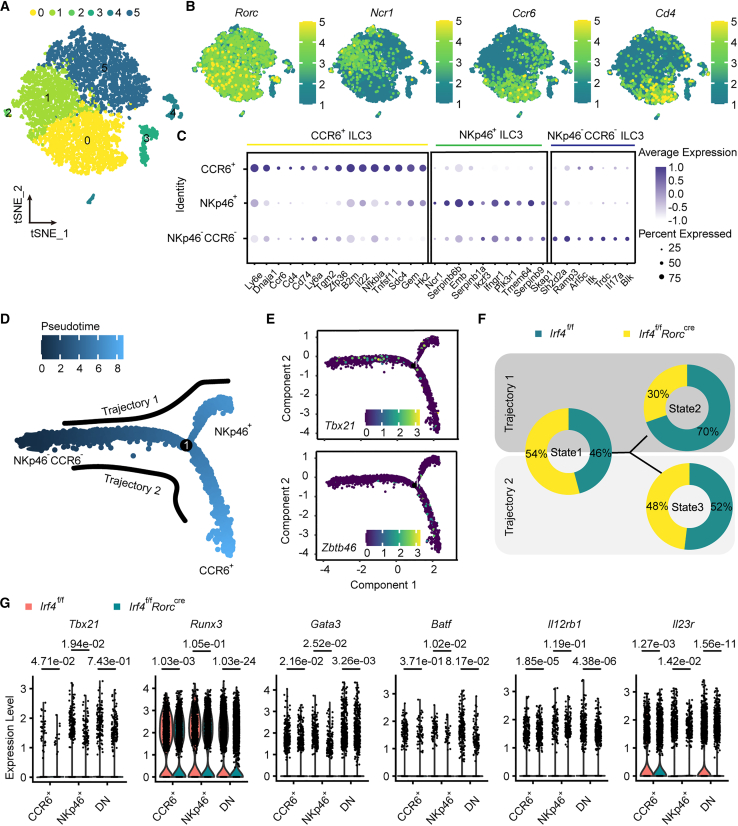
scRNA-seq suggests IRF4 as a sustainer of NKp46^+^ ILC3 phenotype There were 4,424 valid cells (2,353 for *Irf4*^f/f^ mice and 2,071 for *Irf4*^f/f^*Rorc*^cre^ mice), with an average of 1,539 gene transcripts for each cell. (A and B) t-SNE plots visualizing cell state annotations (A) and marker gene expressions (B) in ILC3 cells isolated from *Irf4*^f/f^ and *Irf4*^f/f^*Rorc*^cre^ mice. Four thousand four hundred twenty-four valid cells in total with six clusters (C0–C5, colors indicated). (C) Bubble plot showing the expressions of marker genes across cell states, with the dot size indicating the percentage of marker-positive cells and the color indicating average expression level. (D) Cell developmental trajectory analysis. Trajectory analysis of the single small intestinal ILC3 along with the composition of states. The scale indicates maturity states, ranging from dark blue (representing the least mature) to light blue (representing the most mature). (E) Expression pattern of *Tbx21* and *Zbtb46* along the trajectory. (F) The proportion of different ILC3 subsets between two groups *Irf4*^f/f^ and *Irf4*^f/f^*Rorc*^cre^ along the trajectory. (G) Violin plots visualizing the expression of signature genes. See also Figure S4.

To uncover the underlying mechanism by which IRF4 sustains ILC3 lineage homeostasis, we carried out scRNA-seq on ILC3s harvested from the gut of both *Irf4*^f/f^ and *Irf4*^f/f^ *Rorc*^cre^ mice. Low-quality cells and contaminating cells expressing transcripts of mitochondria were excluded from further analysis. Based on the expressions of reported signature ILC3 markers, we identified all three well-established cell types, namely CCR6^+^, NKp46^+^, and NKp46^−^CCR6^−^ ILC3s (Figures 6A, S4A, and S4B). All clusters belonged to the ILC3 lineage since the gene encoding master transcription factor RORγt was expressed across all clusters (Figure 6B). Cells in cluster 0 displayed high expression of *Ccr6* transcriptional features and are referred to herein as CCR6^+^ ILC3s (Figure 6C). A portion of the cells in these clusters expressed *Cd4*, confirming the close relationship among these cells. Clusters 1 and 2 were identified as NKp46-positive cells according to their high expression of *Ncr1* and *Tbx21*. These cells also showed NKp46-like transcriptional identity genes, including protein-coding gene *Ctsw*, *Socs2,* and cell surface molecules *Ccr*9, as well as cytokine-encoding gene *Ifng*. ILC3s in rest clusters did not express *Ccr6*, *Ncr1*, or other lineage-specific identity genes and likely represented a separate ILC3 lineage. It is important to note that the heterogeneity of ILC3, as reflected by the proportion of clusters within the ILC3 composition, was changed by IRF4 deficiency (Figures S4C and S4D). In particular, WT intestinal NKp46-positive cells clustered differentially in clusters 1–2 (accounting for 30% versus 18% of the cells in IFR4-knockout mice), whereas IRF4-deficient NKp46^−^CCR6^−^ ILC3s were predominant in clusters 3–5 (accounting for approximately 60% compared to 45% of the cells in controls mice). The average percentage of CCR6-positive was similar in the two strains. To investigate the relationship between these clusters, we explored the developmental path across intestinal ILC3s using pseudo-time trajectory analysis rooted in cluster 5 (mainly NKp46^−^CCR6^−^ ILC3s) (Figure 6D). As depicted in the figure, the pseudo-time trajectory showed two unique paths along which WT and KO ILC3s progressed differentially. Progression along trajectory one was accompanied by comparatively higher expression of *Tbx21* along with lower *Zbtb46*^34^ expression that CCR6^+^ ILC3s robustly express, whereas cells on trajectory 2 revealed more intensive expression of *Zbtb46* expression and *Tbx21* could hardly be seen in the tail of trajectory 2 (Figure 6E). Trajectory 1 led through the NKp46^−^CCR6^−^ ILC3s cells toward state two and showed an abundance of WT NKp46^+^ cells (Figure 6F). Trajectory 2 led through the NKp46^−^CCR6^−^ ILC3s cells toward state three, revealing comparable WT and KO CCR6^+^ ILC3 cells.

Analysis of scRNA-seq datasets also revealed the abnormal expression of key transcription factors closely related to NKp46^+^ ILC3s, including downregulation of *Tbx21*, *Runx3*, and *Gata3* (Figure 6G). NKp46^+^ ILC3s' specific gene *Il12rb1* that closely correlated to Notch1 expression^35^ was also declined. Genes related to cytokine secretion, such as Il23r, were also decreased, which was responsible for the formation of functional IL-23 receptor complex. In line with scRNA-seq datasets, flow cytometry confirmed the downregulation of these NKp46^+^ ILC3s-related genes (Figures S4F and S4G). The transcription factor Batf has been found to regulate the homeostasis of ILCs and cytokine secretion of ILC3s.^36^^,^^37^ In addition, data further implicate BATF as a critical epigenetic regulator of MHCII expression on ILC3.^38^ In our data, all ILC3 subsets showed decreased Batf at protein levels. No alteration was observed in the expression level of *Zbtb46* in CCR6^+^ ILC3s compared to the elevated *Tcf7* (Figure S4E).

### IRF4 deficiency compromises MHC-II expression of ILC3 and restrains ILC-mediated apoptosis of effector CD4^+^ T cells

**Figure 7:**
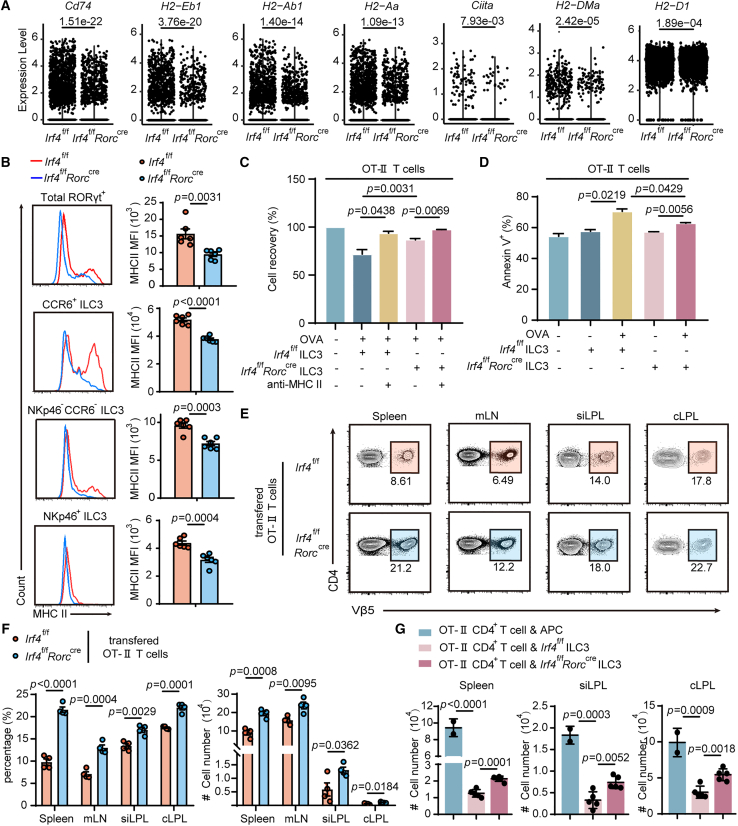
IRF4 deficiency compromises MHC class Ⅱ expression and further restrains ILC-mediated apoptosis of effector CD4^+^ T cells both *in vitro* and *in vivo* (A) Violin plots visualizing the expression of MHC-class-Ⅱ-related signature genes. (B) FACS analysis and MFI of MHC class Ⅱ expression in ILC3s isolated from *Irf4*^f/f^ and *Irf4*^f/f^*Rorc*^cre^ mice (*n* = 6). ILC3 subsets were gated as Lin^−^RORγt^+^ and then CCR6^+^NKp46^−^, CCR6^−^NKp46^+^, or CCR6^−^ NKp46^−^. The lineage cocktail included TCRγδ, CD3ε, CD19, CD5, CD11c, Gr-1, and Ter119. (C and D) Activated OT-Ⅱ CD4^+^ T cells were cultured *ex vivo* with purified ILC3s from the siLP of *Irf4*^f/f^ and *Irf4*^f/f^*Rorc*^cre^ mice in the presence or absence of Ova peptide or the anti-MHC class Ⅱ neutralizing antibody. (C) Quantification of OT-Ⅱ T cells recovery (%). (D) Quantification of Annexin-V^+^ OT-Ⅱ T cells. (E and F) Naive CD4-positive T cells (gating as CD4^+^CD25^−^CD62L^hi^CD44^lo^ cells) were sorted from OT-Ⅱ mice and pre-activated overnight. After pre-activation, CD4 positive T cells were transplanted into recipient *Irf4*^f/f^ and *Irf4*^f/f^*Rorc*^cre^ mice along with OVA peptide administration every 2 days following transfer. Nine days later, mice were sacrificed for further analysis of OT-Ⅱ CD4^+^ T cells (gating as CD8^−^CD4^+^TCRβ^+^Vβ5^+^) transferred in the spleen, mLN, siLPL, and cLPL of recipient mice. (G) One hundred thousand intestinal ILC3s (Lin^−^CD127^+^CD27^−^KLRG1^−^) were sorted from *Irf4*^f/f^ or *Irf4*^f/f^*Rorc*^cre^ mice and transferred with 500,000 activated OT-ⅡCD4^+^ T cells into NCG mice, flowing by OVA peptide i.p. every 2 days. Nine days later, survived OT-ⅡCD4^+^ T cells were quantified. (*n* = 2 APC, *n* = 5 transferred group). Bar graphs are presented as mean ± SEM. A two-tailed Student’s t test was performed for comparisons. The data are representative of at least three independent experiments (B–G). ∗*p* < 0.05, ∗∗*p* < 0.01, ∗∗∗*p* < 0.001. See also Figure S5.

Leveraging the comprehensive transcriptomic coverage of our scRNA-seq data, we systematically evaluated the impact of IRF4 deficiency on CCR6^+^ ILC3 transcriptional programs. Consistent with previous reports demonstrating MHC class II signature enrichment in CCR6^+^ ILC3s (including *CD74*, *H2-Eb1*, *H2-D1*, *H2-Aa*, *Ciita*, and *H2-DMa*), we identified a modest but significant positive correlation between IRF4 and MHC-class II-related gene expression (Figure S5A). IRF4 deficiency induced profound alterations in the MHC class II transcriptional landscape across ILC3 subsets (Figure 7A). Although both total ILC3s and NKp46^−^CCR6^−^ ILC3s showed reduced MHC class II expression, this defect was most pronounced in CCR6^+^ ILC3s (Figure 7B).

The ability of ILC3s to present antigens to T cells has been reported, and both splenic and intestinal ILC3s can process and present peptide antigens by MHC II.^6^^,^^7^^,^^9^ MHC II^+^ ILC3s enriched in the lung-draining lymph nodes have been proven to restrict the expansion of allergen-specific CD4^+^ T cells, demonstrating a pivotal role for antigen-presenting ILC3s in orchestrating immune tolerance.^39^ To further explore the influence of IRF4 deficiency on the function of MHC class Ⅱ^+^ ILC3s, we used *in vitro* ILC3-CD4^+^ T cell co-culture system and evaluated the capacity of ILC3s from littermate controls and IRF4-deficient mice to stimulate CD4 positive T cells *in vitro*. Activated OT-Ⅱ CD4^+^ T cells were co-cultured *ex vivo* with isolated ILC3 cells in the presence or absence of Ova peptide or the neutralizing antibody of anti-MHC class Ⅱ. Co-culture of activated T cells with sorted ILC3s resulted in decreased T cells, which was mitigated in co-culture with MHC class Ⅱ-decreased ILC3s from IRF4-deficient mice and could be rescued by MHC-class-Ⅱ-blocking antibodies (Figure 7C). In the remaining T cells, downregulated cell recovery was associated with increased Annexin V staining (Figures 7D and S5B). Although splenic and small intestinal ILC3s can equally process and present antigens, transcripts related to co-stimulation are only enriched in spleen-derived ILC3s (SP ILC3s). Negligible expression of classical co-stimulatory molecules, like CD40, CD80, and CD86, is observed in intestinal MHC II^+^ RORγt^+^ ILCs.^7^^,^^9^ Researchers proposed that antigen presentation without co-stimulatory factors may limit T cell responses.^40^ These results demonstrated that downregulation of MHC II led to decreased ILC-mediated T cell apoptosis. Hence, different from antigen-presenting ILC3s in the spleen that are efficient in sustaining the maintenance of CD4^+^ memory T cells, intestinal MHC II^+^ ILC3s may be regarded as regulators of CD4^+^ T cell apoptosis.

To investigate the performance of CD4-positive T cells response regulated by MHC class Ⅱ^+^ ILC3s *in vivo*, naive CD4-positive T cells were sorted from OT-Ⅱ mice and transplanted into recipient mice after pre-activation, along with OVA peptide administration. We then quantified these survived OT-Ⅱ T cells transferred in the spleen, mLN, siLPL, and cLPL (Figures 7E and 7F). As speculated, antigen presentation by MHC-II^+^ ILC3s lacking co-stimulatory molecules could lead to T cell apoptosis. We observed that MHC class Ⅱ^+^ ILC3s were also sufficient to influence effector CD4^+^ T cells *in vivo*, and downregulation of MHC-II leads to decreased ILC-mediated T cell apoptosis. Similar results were observed in the NCG mice receiving IRF4-deficient MHC class Ⅱ^+^ ILC3s, as manifested by the significantly higher number of survived T cells after MHC class Ⅱ downregulation (Figure 7G). IRF4 deficiency in ILC3 results in the downregulation of MHC class Ⅱ, which leads to decreased ILC-mediated apoptosis of effector CD4^+^ T cells both *in vivo* and *in vitro*.

### IRF4 directly controls effector genes of both NKp46^+^ ILC3 and MHC class Ⅱ^+^ ILC3s

**Figure 8:**
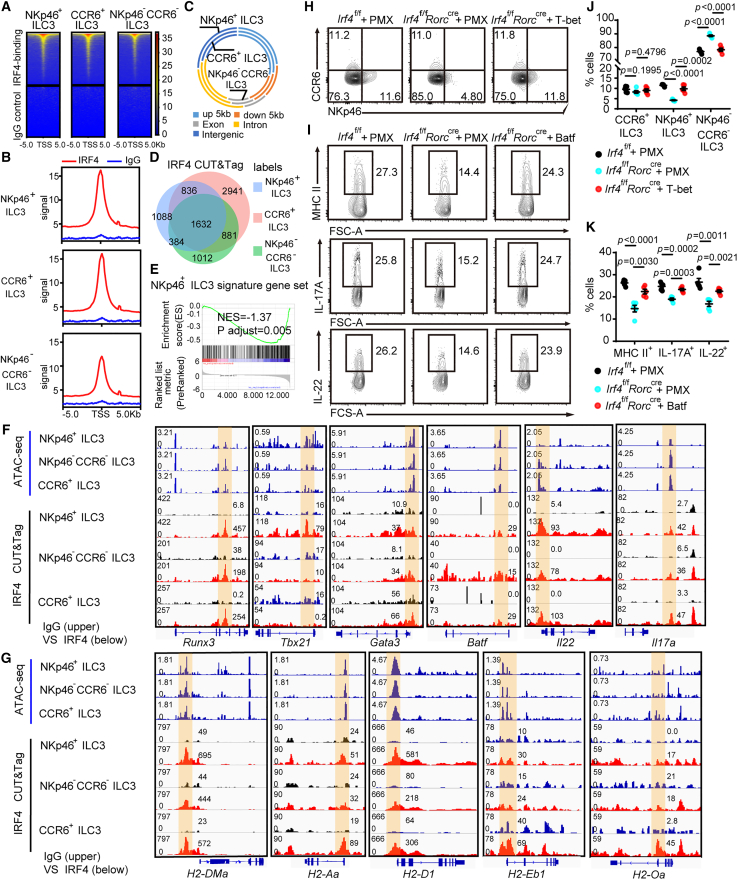
Cleavage under targets and tagmentation sequencing of IRF4 binding in intestinal ILC3 subsets (A) Heatmap showing the genome-wide distribution of IRF4-binding signals at peak centers in ILC3 subsets sorted from *Irf4*^f/f^ and *Irf4*^f/f^*Rorc*^cre^ mice by CUT&Tag. (B) Occupancy of IRF4 at all gene promoter regions (±5 kb of TSS). (C) Donut chart showing the percentages of IRF4 binding at exon regions, intron regions, or intergenic regions. (D) Venn plot displaying the overlap of the IRF4-regulated genes from pairwise comparisons of NKp46^+^ ILC3s, NKp46^−^CCR6^−^ ILC3s, and CCR6^+^ ILC3s. (E) Gene set enrichment analysis (GSEA) of NKp46^+^ ILC3 signature gene sets enriched in shared IRF4-modified genes. (F and G) IGV visualizes the indicated gene locus containing ATAC-seq and IRF4-binding peaks in ILC3 subsets. IRF4 CUT&Tag sequencing data are from two independent replicates. (H–K) Rescue experiments. Retroviruses were generated by transfection of pMX-IRES-GFP plasmids containing the indicated genes into Plat-E cells using PolyJet. CLPs (Lin^−^CD127^+^c-Kit^int^Sca-1^int^Flt3^+^) were sorted from the bone marrow from *Irf4*^f/f and Irf4f/f^ Rorc^cre^ mice and transfected with retroviral supernatants. Retrovirus-transfected CLPs were then collected and adoptively transferred into sublethally irradiated CD45.1^+^ wild-type recipient mice through intravenous tail vein injection. Transduced cells were transferred with CD45.1^+^ wild-type bone marrow cells to help the engraftment of the CLPs. After 2 weeks, recipient mice were sacrificed, and organs were collected for analysis. (H) Flow cytometry of CD45.2^+^GFP^+^ ILC3 subsets isolated from the siLP in the indicated recipient mice (upper). MHC class II expression of CD45.2^+^GFP^+^ ILC3 subsets isolated from the siLP in the indicated recipient mice (lower). (I) Cytokine production in siLP CD45.2^+^GFP^+^ ILC3s from the indicated recipient mice. (J) The percentages of the indicated subsets were compared. (K) The percentages of MHC class II^+^ ILC3s, IL-17A^+^ ILC3s, and IL-22^+^ ILC3s were compared. Bar graphs are presented as mean ± SEM. A two-tailed Student’s t test was performed for comparisons. The data are representative of at least three independent experiments. See also Figure S6.

To identify the target of IRF4 in ILC3 cells and characterize the genome-wide profile of IRF4 binding, we subjected small intestinal ILC3s to chromatin sequencing using cleavage under targets and tagmentation (CUT&Tag). We observed strong enrichment for occupancy by IRF4, but not control immunoglobulin G (IgG), within 5 kb of transcription start sites (TSSs) across the whole genome in ILC3s (Figures 8A and 8B). Most IRF4-binding sites in ILC3 subsets were mainly enriched upstream of promoters (within 5 kb), intergenic regions, and introns (Figure S6A). No alteration was observed in the proportions of peak distribution between ILC3 subgroups (Figure 8C). Then, we overlapped differentially expressed genes that contained IRF4-binding sites among three ILC3 subsets, which may be directly modulated by IRF4 (Figure 8D). Notably, nearly half of the IRF4-binding genes within NKp46^+^ ILC3s were shared by NKp46^−^CCR6^−^ ILC3s, and most of these genes were NKp46 signature genes that were downregulated (Figure 8E). By integrating ATAC sequencing and anti-IRF4 CUT&Tag sequencing datasets, we found these gene loci also contained ATAC-seq peaks representing areas of accessible chromatin in all ILC3 subsets (Figures 8F and S6B). IRF4-binding peaks within genes of transcription factors such as *Runx3*, *Tbx21*, and *Gata3* loci were observed in all ILC3 subpopulations. We also found IRF4-binding peaks shared by all three subsets at the *Batf* locus (Figure 8F). IRF4 directly bound the *Il22*, *Il17a*, *Il23r*, and *Il12rb1* loci, but not IgG, further demonstrating that IRF4 serves direct roles in regulating ILC3s signature identity gene expression.

We compared individual patterns of IRF4-binding peaks and ATAC-seq peaks in multiple MHC class Ⅱ signature identity genes to clarify the underlying mechanism of MHC class Ⅱ downregulation in IRF4-deficient mice (Figure 8G). Among these transcripts associated with MHC class Ⅱ expression, the *H2-DMa*, *H2-Aa*, *H2-D1*, *H2-Eb1*,and *H2-Oa* loci were directly bound by IRF4, further indicating that IRF4 serves direct roles in the regulation of MHC class Ⅱ signature genes expression, although *CIITA* loci did not exhibit apparent IRF4-binding peaks (Figure S6B). Of note, different from *H2-DMa* and *H2-Aa*, the IRF4-binding peaks in *H2-D1*, *H2-Eb1*, and *H2-Oa* were more enriched in CCR6^+^ ILC3 than in the other two subgroups. These findings demonstrated that IRF4 may modulate the expression of MHC-Ⅱ in ILC3 by directly binding to the locus of transcripts responsible for MHC-Ⅱ expression.

To ascertain the transcription factors that associate with IRF4 to regulate ILC3s, we initiated a motif enrichment analysis of the shared peaks within NKp46^+^ ILC3s, NKp46^−^CCR6^−^ ILC3s, and CCR6^+^ ILC3s in the anti-IRF4 CUT&Tag dataset. Both Batf and Tbx21 emerged as notably enriched transcription factors associated with IRF4 (Figure S6C). To further explore the role played by these factors in IRF4-deficient ILC3s, we infected CLPs from *Irf4*^f/f^ *Rorc*^cre^ mice with Batf and T-bet retroviral vectors, providing long-term modulation of gene expression in infected cells and their progeny. Retrovirus-transfected CLPs were transferred into sublethally irradiated CD45.1^+^ recipient mice that were sacrificed 2 weeks later for analysis. The results showed that T-bet overexpression restored NKp46^+^ and DN ILC3s populations in IRF4-deficient cells, confirming T-bet as a critical downstream effector of IRF4 (Figures 8H and 8J). In addition, the overexpression of Batf potently increased MHC class II expression in ILC3s relative to that in mock-transfected control. Notably, IRF4-deficient ILC3s derived from mock-transfected CLPs produced minimal IL-22 and IL-17A, whereas transfection with Batf led to high levels of production (Figures 8I and 8K).

## Discussion

Recent comparative transcriptome analyses have elucidated the roles of core transcriptional factors in maintaining the homeostasis and functionality of NKp46^+^ ILC3s. Among the three RUNX family members, Runx3 was predominantly expressed in both ILC1 and ILC3 subsets. Conditional deletion of Runx3 in NKp46-expressing cells led to a significant decrease in intestinal NKp46^+^ ILC3 populations. The graded expression of T-bet governs the differentiation of CCR6-negative ILC3s and serves as a molecular switch regulating the ILC3-to-ILC1 conversion. Insufficient T-bet expression eventually renders NKp46^−^CCR6^−^ ILC3s less prone to transition into NKp46^+^ ILC3s. Although the level of GATA-3 was relatively low in ILC3, its critical role in committed ILC3s has been well established. Genetic ablation of GATA-3 blocks the further conversion from NKp46^−^CCR6^−^ ILC3s into NKp46-positive ILC3 cells. In this study, we identify IRF4 as a novel essential regulator within the transcriptional network governing NKp46^+^ ILC3 development. Decreased expression of Runx3, Gata3, and T-bet were observed in the absence of IRF4. Validating our scRNA-seq data, these NKp46^+^-ILC3-associated transcription factors were downregulated, concomitant with T-bet loss, ultimately impairing the transition of NKp46^−^CCR6^−^ ILC3s to NKp46^+^ ILC3s.

Beyond its essential roles in ILC3 homeostasis, IRF4 critically regulates the production of IL-22 and IL-17A. In T-cell-dependent metastatic colitis models, IIRF4 deficiency conferred protection against inflammation, correlating with reduced RORα/γt expression and impaired IL-17A and IL-22 secretion.^41^ IRF4 promotes IL-17 expression in mucosal tissues through direct binding to the IL-17 promoter, thereby modulating Th17-mediated colitis. The observed cytokine secretion defects in ILC3s likely reflect this IRF4-dependent regulatory mechanism. Our ATAC-seq and anti-IRF4 CUT&Tag sequencing analyses revealed direct IRF4 binding at the *Il22* and *Il17a* loci (compared to IgG controls), providing definitive evidence that IRF4 directly regulates the expression of ILC3 signature identity genes. Furthermore, IRF4-deficient mice exhibited downregulation of IL-17A-associated genes, including the cell surface receptor IL-23R responsible for functional IL-23 receptor complex,^42^ potentially explaining the observed reduction in IL-17A production.

Notably, ILC3s also participate in adaptive immune regulation through direct crosstalk with T cells. Emerging evidence demonstrates that ILC3s, particularly LTi or LTi-like cells (CCR6^+^ ILC3s), expressing MHC class II molecules can present antigens to CD4^+^ T cells.^7^^,^^8^ IRF4 has been known to regulate the expression of costimulatory molecules (CD80/CD86) and MHC-class-Ⅱ-dependent antigen presentation in multiple immune cell types, including dendritic cells,^43^^,^^44^ CD226^+^ macrophages,^45^ and lymphoma B cells.^46^ In this study, we observed that IRF4 deficiency in ILC3s disrupted the MHC-class-II-associated transcriptional program and significantly reduced MHC class II expression in CCR6^+^ ILC3s.These findings suggest that ILC3 may induce immune tolerance in the intestine through ILC3-mediated antigen presentation. MHC class Ⅱ^+^ ILC3s have been proven to maintain immune tolerance to the microbiota and intestinal health via inducing the balance between microbiota-specific RORγt^+^ Treg cells and TH17 cells.^47^ Of note, some transcription factors have been implicated in controlling MHC class Ⅱ^+^ ILC3 function, including BATF,^38^ ZBTB46,^34^ and TCF-1.^48^ Regarding their antigen-presenting capacity, the transcriptional repressor ZBTB46 robustly expressed by CCR6^+^ ILC3s can affect the acquisition of activating or tolerogenic properties in MHC-Ⅱ^+^ ILC3s. BATF serves a pivotal role in regulating the functional plasticity of intestinal ILC3s, with BATF deficiency leading to enhanced activation of intestinal CD4^+^ T cells.^38^

Here, our multi-omics analyses reveal that IRF4 interacts with regulatory regions of genes critical for ILC3 function, including *Il17a*, *Il22*, *Tbx21*, *Batf*, and MHC II genes (*H2-DMa*, *H2-Aa*, *H2-D1*, *H2-Eb1*, *H2-Oa*), suggesting IRF4 as a direct transcriptional regulator. IRF4 binds the Batf locus, initiating a transcriptional hierarchy where BATF amplifies MHC II expression. BATF overexpression restores MHC II levels in IRF4-deficient ILC3s, demonstrating its role as a downstream mediator. Motif enrichment analysis also revealed Batf as a significantly enriched transcription factor associated with IRF4. Interestingly, functional assays also showed that BATF overexpression restores IL-22 and IL-17A production in IRF4-deficient cells, indicating potential cooperative mechanisms between these transcription factors. IRF4 and BATF cooperatively regulate genes critical for ILC3 function. Collectively, IRF4 exerts its regulatory effects on these genes through both direct transcriptional regulation and an indirect regulatory network involving Batf.

### Limitations of the study

Although this study establishes IRF4 as a critical regulator of intestinal ILC3 homeostasis and function, several avenues for further investigation remain. First, the precise mechanistic interplay between IRF4 and other transcription factors (e.g., BATF, T-bet, Runx3, GATA-3) identified in the regulatory network requires deeper exploration. Although the study demonstrates direct binding of IRF4 to *Batf* and *Tbx21* loci and partial rescue of defects via their overexpression, the hierarchical or cooperative relationships among these factors—especially in the context of MHC class II^+^ ILC3 subsets—remain incompletely characterized. Second, the functional consequences of IRF4-mediated MHC class II downregulation in ILC3s, such as its impact on antigen presentation to specific T cell subsets (e.g., regulatory T cells vs. effector T cells) *in vivo*, were not fully dissected. Additionally, while mouse models were used to characterize IRF4 deficiency, whether these findings translate to human ILC3 biology—particularly in the context of intestinal inflammatory diseases or infections—warrants further validation.

## Resource availability

### Lead contact

Further information and requests for resources and reagents should be directed to and will be fulfilled by the lead contact, Lie Wang (wanglie@zju.edu.cn).

### Materials availability

This study did not generate new unique reagents.

### Data and code availability

• CUT&Tag and scRNA-seq datasets generated in this study have been deposited in the Gene Expression Omnibus (GEO) database under the accession number GSE256436 (CUT&Tag) and GSE271288 (scRNA-seq).
• The original code of sequencing data used in this study is available in the GitHub repository at https://github.com/anjin8023/wanglab.git.
• Any additional information required to reanalyze the data reported in this paper is available from the lead contact upon reasonable request.

## Acknowledgments

We thank Prof. Ju Qiu (Shanghai Institutes for Biological Sciences) for her generous gifts of *C. rodentium*. We thank Prof. Guanghua Huang (Fudan University) for his generous gifts of *C. albicans*. We thank Yanwei Li, Yingying Huang, Wei Yin, Jiajia Wang, Chun Guo, Nan Zhou, Xiaomin Yu, and Hongying Shen from the Core Facilities, Zhejiang University School of Medicine, for their technical support. We thank Yanxia Ding, Huihui Jin, and Xuliang Zhang from Animal Facilities, Zhejiang University, for mice maintenance. Graphic abstract was created in BioRender. An, J. (2025) https://BioRender.com/cnwzy9j. This work was supported by grants from the 10.13039/501100012166National Key R&D Program of China (2023YFA1800202 and 2024YFF0728703), the 10.13039/501100001809National Natural Science Foundation of China (32341002 and 32030035), Science and Technology Innovation 2030-Major Project (2021ZD0200405), and the 10.13039/501100012226Fundamental Research Funds for the Central Universities (226-2024-00161).

## Author contributions

L.W., L.L., and D.W., supervision, conceptualization, project administration, and writing—review and editing. L.L. and D.W., writing—review and editing. L.W., funding acquisition. X.G., investigation, methodology, project administration, and writing—original draft. X.G., X.S., and Q.X: data curation and formal analysis. Y.Z., L.D., S.H., H.J., and Q.W., investigation and methodology.

## Declaration of interests

The authors declare that the research was conducted in the absence of any commercial or financial relationships that could be construed as a potential conflict of interest.

## STAR★Methods

### Key resources table

**Table:** 

REAGENT or RESOURCE	SOURCE	IDENTIFIER
**Antibodies**		

Biotin CD3e (clone 145-2C11)	eBioscience	Cat#36-0031-85; RRID: AB_469747
Biotin TCR gamma/delta (clone GL3)	eBioscience	Cat#13-5711-85; RRID: AB_466669
Biotin Ly-6G/Ly-6C (clone RB6-8C5)	eBioscience	Cat#13-5931-82; RRID: AB_466800
Biotin TER-119 (clone TER-119)	Biolegend	Cat#116203; RRID: AB_313704
Biotin CD19 (clone 1D3)	eBioscience	Cat# 13-0193-86; RRID: AB_657655
Biotin CD5 (clone 53-7.3)	Biolegend	Cat#100603; RRID: AB_312732
Biotin B220 (clone RA3-6B2)	Biolegend	Cat#10320; RRID:AB_312988
Biotin anti-mouse NK-1.1 (clone PK136)	Biolegend	Cat#108703; RRID: AB_313390
Biotin anti-mouse CD11c (clone N418)	Biolegend	Cat#117303; RRID: AB_313772
Biotin anti-mouse/human CD11b (clone M1/70)	Biolegend	Cat#101203; RRID: AB_312786
PE-Cyanine7 anti-CD27 (clone LG.7F9)	eBioscience	Cat#25-0271-82; RRID:AB_1724035
PE Anti-Mouse IL-17A (TC11-18H10)	BD Biosciences	Cat#561020; RRID: AB_397256
PE anti-mouse CD45.1 (A20)	Biolegend	Cat# 110707; RRID: AB_313496
PE anti-Mouse IFN-γ (XMG1.2)	BD Biosciences	Cat# 554412; RRID: AB_395376
PerCP-Cy™5.5 Mouse anti-Ki-67 (B56)	BD Biosciences	Cat# 561284; RRID: AB_10611574
APC-Cy™7 Mouse Anti-Mouse NK-1.1 (PK136)	BD Biosciences	Cat# 560618; RRID: AB_1727569
APC Mouse anti-Mouse CD45.2 (104)	BD Biosciences	Cat# 561875; RRID: AB_1645215
BV421 Mouse Anti-Mouse RORγt (Q31-378)	BD Biosciences	Cat# 562894; RRID: AB_2687545
PE CD127 Monoclonal Antibody (A7R34)	eBioscience	Cat# 12-1271-82; RRID: AB_465844
APC IL-22 Monoclonal Antibody (IL22JOP)	eBioscience	Cat# 17-7222-82; RRID: AB_10597583
PE-Cyanine7 KLRG1 Monoclonal Antibody (2F1)	eBioscience	Cat# 25-5893-82; RRID: AB_1518768
APC-Cyanine7 c-Kit Monoclonal Antibody (2B8)	Invitrogen	Cat# A15423; RRID: AB_2534436
PE/Cyanine7 anti-mouse CD196 (29-2L17)	Biolegend	Cat# 129815; RRID: AB_1877244
PE Mouse IgG1 kappa Isotype Control (P3.6.2.8.1)	eBioscience	Cat# 12-4714-82; RRID: AB_470060
APC anti-mouse CD8a (53-6.7)	Biolegend	Cat# 100711; RRID: AB_312750
PE anti-mouse PLZF Antibody (9E12)	Biolegend	Cat# 145803; RRID: AB_2561966
APC anti-mouse CD335 (NKp46) (29A1.4)	Biolegend	Cat# 137607; RRID: AB_10612749
APC/Cyanine7 anti-mouse CD4 (GK1.5)	Biolegend	Cat# 100413; RRID: AB_312698
BV650 Streptavidin	BD Biosciences	Cat# 563855; RRID: AB_2869528
APC anti-mouse LPAM-1 (Integrin α4β7) (DATK32)	Biolegend	Cat# 120607; RRID: AB_10719833
PE anti-mouse Ly-6A/E (Sca-1) (D7)	Biolegend	Cat# 108107; RRID: AB_313344
PE-Cyanine5 CD135 (Flt3) Monoclonal Antibody (A2F10)	eBioscience	Cat# 15-1351-82; RRID: AB_494219
PE Gata-3 Monoclonal Antibody (TWAJ)	eBioscience	Cat# 12-9966-42; RRID: AB_1963600
PE-Cyanine7 EOMES Monoclonal Antibody (Dan11mag)	eBioscience	Cat# 25-4875-82; RRID: AB_2573454
PE AHR Monoclonal Antibody (4MEJJ)	eBioscience	Cat# 12-5925-82; RRID: AB_2572644
PE anti-IRF4 Antibody (IRF4.3E4)	Biolegend	Cat# 646403; RRID: AB_2563004
PE Mouse Anti-RUNX3	BD Biosciences	Cat# 564814; RRID: AB_2738969
PE IRF4 Monoclonal Antibody (3E4)	eBioscience	Cat# 12-9858-82; RRID: AB_10852721
PE anti-mouse IL-23R Antibody (12B2B64)	Biolegend	Cat# 150903; RRID: AB_2572188
PE anti-T-bet Antibody (4B10)	eBioscience	Cat# 644809; RRID: AB_2028583
PE-conjugated anti–IL-12Rβ1	R and D Systems	Cat# FAB1998P; RRID: AB_10571374
IRF-4 Rabbit mAb (D9P5H)	Cell Signaling	Cat# 15106T; RRID: AB_2798709
Anti-BATF (EPR21911)	Abcam	Cat# ab221146; RRID: AB_3665801
Alexa Fluor™ 594 Donkey Anti-Rabbit IgG (H + L)	Cell Signaling	Cat# A21207; RRID: AB_2340621
Normal Rabbit IgG (2729)	Cell Signaling	Cat# 2729S; RRID:AB_1031062
Anti-Rabbit IgG (H + L)	Sigma-Aldrich	Cat# SAB3700883; RRID: AB_3697514

**Bacterial and virus strains**		

DH5α Chemically Competent Cell	Tsingke Biotech	Cat#TSC-C14
*Citrobacter rodentium* strain DBS100	ATCC	Stock# 51459
*Candida albicans* strain SC5314.	Guanghua Huang Lab, Fudan University	Gifted from Prof. Guanghua Huang

**Chemicals, peptides, and recombinant proteins**		

Fixable Viability Dye 450	eBioscience	Cat#65-0863-14
Fixable Viability Stain 510	BD	Cat#564406
FBS	Gibco	Cat#10270
PMA	Sigma-Aldric	Cat# P1585
DTT, Molecular Grade (Dry Powder)	Promega	Cat#V3151
Ionomycin	Sigma-Aldric	Cat# I3909
Polybrene	Millpore	Cat# TR-1003-G
DNase I	Sigma	Cat# DN25
Collagenase VIII	Sigma Aldrich	Cat#C2139
Percoll	Cytiva	Cat#17-0891-01
PrimeSTAR MAX Premix	TAKARA	Cat# R045A
Mouse IL-23 Recombinant Protein	Peprotech	Cat# 14-8231-63
IL-1β	Peprotech	Cat# SRP8033
Interleukin-7	Peprotech	Cat# I4892
IL-6	Peprotech	Cat# SRP3330
Murine SCF	Peprotech	Cat#250-03-50
RetroNectin	Takara	Cat#T100A/B

**Critical commercial assays**		

BD Rhapsody™ WTA Amplification Kit	BD Biosciences	Cat# 633801
AMPure® XP magnetic beads	Beckman	Cat# A63880
Qubit™ dsDNA HS Assay Kit	Thermo Fisher Scientific	Cat# Q32851
BD Rhapsody™ Cartridge Reagent Kit	BD Biosciences	Cat# 633731
BD Rhapsody™ cDNA Kit	BD Biosciences	Cat# 633773

**Deposited data**		

scRNA-seq	This paper	GSE271288
CUT&Tag sequencing	This paper	GSE281331

**Experimental models: Cell lines**		

HEK 293T	ATCC	Cat#ACS-4500
Plat E	Shanghai Institutes for Biological Sciences	Gifted from Prof. Xiaolong Liu

**Experimental models: Organisms/strains**		

Mouse:*Irf4*^*flox/flox*^	Jackson Laboratories	Strain #:009380 RRID:IMSR_JAX:009380
Mouse:Rorc-cre	Jackson Laboratories	Strain #: 022791 RRID:IMSR_JAX:022791
Mouse: NCG	GemPharmatech (Nanjing, China)	Strain #:T001475
Mouse:CD45.1	Jackson Laboratories	Strain #: 002014 RRID:IMSR_JAX:002014
Mouse:OT-II	Jackson Laboratories	Strain #:004194 RRID:IMSR_JAX:004194

**Oligonucleotides**		

RORγt-cre genotyping. Forward: GGAAAATGCTTCTGTCCGTTTG	Tsingke Biotech	N/A
RORγt-cre genotyping. Reverse: TTGGTCCAGCCACCAGCTTG	Tsingke Biotech	N/A
IRF4 genotyping. Common: AAT ACT GAG CTG CAG TCT AGC	Tsingke Biotech	N/A
IRF4 genotyping. Wild type Reverse: TGT TGC TGG TGG AGA GGA AG	Tsingke Biotech	N/A
IRF4 genotyping. Mutant Reverse: GAC CAC TAC CAG CAG AAC AC	Tsingke Biotech	N/A

**Recombinant DNA**		

pMX-IRES-tdTomato	This paper	N/A

**Software and algorithms**		

GraphPad Prism v8	GraphPad	https://www.graphpad.com/guides/prism/8/userguide/tips_for_using _prism.htm
FlowJo v10	TreeStar	https://www.flowjo.com/solutions/flowjo/do wnloads
R version 4.0.2	R Core	https://www.R-project.org/
Adobe Illustrator	Adobe	https://www.adobe.com/cn/

**Other**		

Dynabeads® Biotin Binder	Thermo Fisher Scientific	Cat#11047

### Experimental model and study participant details

#### Mice

The *Irf4*^f/f^ mouse strain was bought from the Jackson Laboratory (Bar Harbor, ME). The *Rorc*-cre tool mice (JAX: 022791) were kindly provided by Prof. Ju Qiu (Shanghai Institutes for Biological Sciences, Chinese Academy of Sciences). *Irf4*^f/f^ mice were crossed to *Rorc*-cre mice to obtain respective conditional knockout mice. We purchased NOD Prkdc^em26Cd52^Il2rg^em26Cd22^/Nju (NCG, T001475) mice from the Nanjing Biomedical Research Institute of Nanjing University. The OT-Ⅱ (JAX: 004194) TCR transgenic mice were from Jackson Laboratory. CD45.1 mice (2 months old) were obtained from Zhejiang University Laboratory Animal Center. Mice at 6–8 weeks of age were sacrificed for all experiments unless otherwise noted. The Institutional Animal Care and Use Committee approved all animal procedures, and all mice were bred and maintained under specific pathogen-free conditions at the Zhejiang University Laboratory Animal Center.

### Method details

#### Isolation of intestinal lamina propria lymphocytes (LPLs)

LPLs were isolated from the small intestine lamina propria (siLP) as previously described.^49^ Small intestines were dissected, and fat tissues and Peyer’s patches were meticulously removed. Intestines were dissected longitudinally and cut into 5mm-long pieces, subsequently washed with DMEM. Following this, the intestinal pieces were incubated for 10 min with constant agitation in DMEM containing 3% fetal bovine serum (FBS), 0.2% Hanks, 0.5 M EDTA, and dithiothreitol (0.145 mg/mL). All intestinal pieces were filtered and transferred to DMEM containing 0.5 M EDTA and 1 M HEPES and incubated for about 10 min. The tissues were then digested using DNase I (Sigma, 50 mg/mL) and collagenase Ⅱ (Worthington Biochem, 145 mg/mL) at 37°C for 5 min, and this step was repeated twice. The dissociated cells were filtered with a 100-μm cell strainer and harvested from the interphase of an 80%–40% Percoll (GE Healthcare) gradient. After being washed with PBS, LPLs were collected for further analysis.

#### Cell stimulation and flow cytometry

To assess cytokine secretion, *ex vivo* LPLs were resuspended in DMEM supplemented with 10% FBS and then stimulated for 4 h at 37°C with IL-23 (PeproTech, 40 ng/mL) and IL-1β (PeproTech, 20 ng/mL) for IL-22 production, or with PMA (Sigma-Aldrich, 50 ng/mL) and ionomycin (Sigma-Aldrich, 1 mg/mL) for IL-17A production. A protein transport inhibitor, brefeldin A (BFA, 1000×, Invitrogen), was added to block cytokine secretion 0.5 h after stimulation.

Dead cells were labeled using Fixable Viability Dye (eBioscience), and Fc receptor blockade was performed with anti-CD16/32 (clone 93, Biolegend). For the detection of bone marrow precursor cells, lineage markers included antibodies against TCRγδ, CD3ε, CD19, B220, NK1.1, CD11b, CD11c, Gr-1, and Ter119. Peripheral ILCs were identified using the lineage cocktail containing TCRγδ, CD3ε, CD19, CD5, Gr-1, CD11C and Ter119. When intracellular transcription factor or cytocine staining was carried out, the cells were fixed and permeabilized with an Foxp3/transcription factor staining buffer set according to the manufacturer’s instructions (Invitrogen) after staining for surface markers. Flow cytometry was performed with a BD Fortessa (BD Biosciences), and data were analyzed using FlowJo10 software. Cell sorting was performed by an FACSAria Ⅱ flow cytometer and Moflo Astrios EQ (Beckman).

The following antibodies were purchased from eBioscience, BD Biosciences, or Biolegend: CD4 (RM4-5), CD8α (53-6.7), IL-17A (TC11-18H10), TCR-β (H57-597), CD27 (LG.7F9), CD25 (PC61.5), NK1.1 (PK136), IL-22 (IL22JOP), Eomes (Dan11mag), PLZF (B263557), CD45.2 (104), Ki67 (B56), RORgt (Q31-378), Flt3 (A2F10), NKp46 (29A1.4), KLRG1 (2F1), GATA3 (TWAJ), CD127 (A7R34), CD117 (2B8), CCR6 (29-2L17), B220 (RA3-6B2), Sca-1 (D7), Isotype (P3.6.2.8.1), IgM (II/41), α4β7 (DATK32), CD45.1 (A20), IRF4 (B256112) and Streptavidin. Apoptosis was measured using the Annexin V-FITC Apoptosis Kit (Lianke).

#### Bone marrow chimeras

To generate bone marrow chimeras, CD45.2^+^ bone marrow cells from *Irf4*^f/f^ and *Irf4*^f/f^ *Rorc*^cre^ mice were mixed with CD45.1^+^ wild-type bone marrow cells in a 1:1 ratio. The mixture was intravenously injected into sublethally irradiated (7Gy) CD45.1^+^ wild-type recipient mice. Eight weeks after cell transfer, intestinal ILC3s derived from CD45.2^+^ donor cells were analyzed by flow cytometry.

#### *In vitro* coculture assay

Naive CD4-positive T cells (gating as CD8^−^CD4^+^CD25^−^CD62L^hi^CD44^lo^ cells) were sorted from OT-II mice and pre-activated overnight with 1ug/ml soluble purified anti-CD3 (16-0031-85; Invitrogen) and anti-CD28 (16-0281-85; Invitrogen). Activated CD4 positive T cells (CD5^+^CD4^+^CD3^+^, 10x10^3^ per well) were purified and co-cultured sorted ILC3 cells (5x10^3^ per well) from the small intestine of *Irf4*^f/f^ *Rorc*^*Cre*^ mice or wild-type mice in the presence or absence of Ova peptide (OVA 323–339, ISQAVHAAHAEINEAGR, 10ug/ml) or the neutralizing antibody of anti-MHCII (1ug/ml). After 72 h of culture, further analysis was carried out.

#### T cell adoptive transfer

Naive CD4-positive T cells (gating as CD8^−^CD4^+^CD25^−^CD62L^hi^CD44^lo^ cells) were sorted from OT-II mice and pre-activated overnight with CD3 (1ug/ml) and CD28 (1ug/ml). After pre-activation, purified CD4 positive T cells (CD5^+^CD4^+^CD3^+^) were transplanted into recipient *Irf4*^f/f^ *Rorc*^cre^ and wild-type mice along with OVA peptide administration (50μg) every 2 days following transfer. Nine days later, mice were sacrificed for further analysis of OT-Ⅱ CD4^+^ T cells transferred in the spleen, mLN, siLPL, and cLPL of recipient mice.

ILC3s (Lin^−^CD127^+^CD27^−^KLRG1^-^) were sorted from *Irf4*^f/f^ or *Irf4*^f/f^ *Rorc*^cre^ mice. Purified ILC3s were pulsed for 3 h in complete media with OVA peptide (10μg/ml). One hundred thousand pre-pulsed ILC3s or APCs were transferred with five hundred thousand activated OT-Ⅱ CD4^+^ T cells into NCG mice, flowing by OVA peptide i.p. every 2 days. Nine days later, survived OT-Ⅱ CD4^+^ T cells were quantified.

#### *C. rodentium* infection

*Irf4*^f/f^ and *Irf4*^f/f^ *Rorc*^cre^ mice were orally inoculated with 5 × 10^9^ CFUs of *C. rodentium* (DBS100), after being treated with ABX (autoclaved water supplemented with antibiotics: ampicillin 1 g/L, gentamicin 1 g/L, metronidazole 1 g/L, and vancomycin 0.5 g/L) treatment for 1 week. Body weight was monitored for seven days, and mice were sacrificed on day 7. Feces was weighed and plated on MacConkey agar plates to determine bacterial colonies. The small intestines were used for lymphocyte isolation, and analyzed for IL-22 production. The colons were measured for lengths and fixed for 24 h in 4% methanol for H&E staining. For histological scoring, colonic tissue sections were blindly graded on a scale of 0–5 for each of the following parameters: (a) epithelial lesions (crypt elongation, hyperplasia, erosion, and ulceration/necrosis), (b) mural inflammation, and (c) edema for an overall maximal total histology score of 15.

#### *C. albicans* infection

*C. albicans s*train SC5314 was cultured in YPD (yeast nitrogen base with 2% glucose, 100 μg/mL ampicillin, 0.01 mg/mL vancomycin, 0.1 mg/mL gentamicin). *C. albicans* (1 × 10^8^ CFUs) was administered to *Irf4*^f/f^ and *Irf4*^f/f^ *Rorc*^cre^ mice by gavage after 3-day ABX-1 (autoclaved water supplemented with streptomycin 2 mg/mL, fluconazole 0.2 mg/mL and gentamicin 0.2 mg/mL) treatment. ABX-I was replaced with ABX-II (Streptomycin 2 mg/mL, Gentamicin 0.2 mg/mL, and Ampicillin 2 mg/mL) on day 3 and lasted until the end of the model. Body weight changes were monitored in the following seven days, and all mice were sacrificed on day 11. Subsequent steps were consistent with the *C. rodentium* model, except for the identification of IL-17A expression. For histological scoring, colonic tissue sections were blindly graded on a scale of 0–5 for each of the following parameters: (a) epithelial lesions (crypt elongation, hyperplasia, erosion, and ulceration/necrosis), (b) mural inflammation, and (c) edema for an overall maximal total histology score of 15.

#### Cell adoptive transfer into NCG mice

NCG mice were adoptively transferred with eighty thousand intestinal ILC3s (Lin^−^CD127^+^CD27^−^KLRG1^-^) sorted from *Irf4*^f/f^ and *Irf4*^f/f^ *Rorc*^cre^ mice or PBS as control after treated with ABX for 1 week. ILC3s were stimulated with IL-23 and IL-1β for 30 min before being injected into NCG mice through the tail vein. NCG mice were orally inoculated with *C. rodentium* 24 h after adoptive transfer. Body weight was monitored for 9 days, and all mice were sacrificed for further analysis on day 9.

NCG mice were adoptively transferred with eighty thousand intestinal ILC3s sorted from *Irf4*^f/f^ and *Irf4*^f/f^ *Rorc*^cre^ mice or PBS as control after 3-day ABX-1 treatment. ILC3s were stimulated with PMA and ionomycin for 30 min before injected to NCG mice through the tail vein. ABX-I was replaced with ABX-II at day 3 and lasted until the end of the model. *C. albicans* was administered to NCG mice by gavage 24 h after adoptive transfer. Body weight was monitored for 9 days, and all mice were sacrificed for further analysis at day 9.

#### Histology

Colons were removed intact, fixed in 4% paraformaldehyde and embedded in paraffin, followed by sectioning and staining with hematoxylin and eosin according to standard laboratory procedures.

#### Gene function of ILCs for *in vivo* analysis using retroviral transfection

Retroviruses were generated by transfection of pMX-IRES-GFP plasmids containing the indicated genes into Plat-E cells using PolyJet (SignaGen). Media were replaced 12/18 h after transfection, and retroviral supernatants were collected after 48 h. Then, 48-well plates were coated with RetroNectin (TaKaRa, 25 μg mL^−1^) overnight at 4°C. After blocking with BSA and washing, 1 mL of supernatant was added and followed by centrifugation for 2 h at 1,500g at 32°C.

The bone marrow from *Irf4*^f/f^ and *Irf4*^f/f^ Rorc^cre^ mice was aspirated to create a single-cell suspension.^50^ CLPs (Lin^−^CD127^+^c-Kit^int^Sca-1^int^Flt3^+^) were enriched by Dynabeads Biotin Binder (Invitrogen) after lineage staining and then superficially stained and sorted. We resuspended CLPs in CLP medium (αMEM medium containing 10% FBS, penicillin–streptomycin, 1× non-essential amino acids, 1 mM sodium pyruvate, 2 mM L-glutamine, 20 mM HEPES and 50 μM β-ME) and added 100,000 cells per well in the presence of IL-7 (20 ng mL^−1^), IL-6 (10 ng mL^−1^), SCF (100 ng mL^−1^), Flt3L (20 ng mL^−1^), TPO (10 ng mL^−1^) and polybrene (Merck, 5 μg mL^−1^). Ten hours later, retrovirus-transfected CLPs were collected and adoptively transferred into sublethally irradiated CD45.1^+^ wild-type recipient mice through intravenous tail vein injection. Transduced cells were transferred with CD45.1^+^ wild-type bone marrow cells to help the engraftment of the CLPs. After 2 weeks, recipient mice were sacrificed, and organs were collected for analysis.

#### CUT&Tag and analysis

H3K4me3 and Irf4 ChIP of three subsets of ILC3s were both performed on 100,000 cells as previously described according to the protocol of Hyperactive *In-Situ* ChIP Library Prep Kit for Illumina (TD 901, Vazyme).^51^^,^^52^ Briefly, the sorted cells were washed with 500 μL wash buffer supplemented with 1× protease inhibitor cocktail (Sigma-Aldrich, 5056489001). Cell pellets were resuspended in the wash buffer. After two washes with binding buffer, Concanavalin A-coated magnetic beads were added and incubated at room temperature. Bead-bound cells were resuspended in 50 μL of antibody buffer. 1 μg of H3K4me3 antibody (Active Motif, 39016), Irf4 (CST, D9P5H), or Normal Rabbit IgG (CST, 2729) were added. The mixture was incubated overnight at 4°C with gentle rotation. After removing the primary antibody, the secondary antibody (goat anti-rabbit IgG, Sigma-Aldrich, SAB3700883) diluted in 50 μL of Dig-wash buffer at a ratio of 1:100 was added and incubated at room temperature. The cells were then incubated with 0.04 μM Hyperactive pG-Tn5 Transposase diluted in Dig-300 buffer at room temperature for 1 hour with slow rotation. Finally, the cells were resuspended in tagmentation buffer and incubated at 37°C for 1 h. DNA was purified by phenol-chloroform-isoamyl alcohol extraction and ethanol precipitation after terminating tagmentation. DNA library amplification was performed according to manufacturer’s instructions and washed with VAHTS DNA Clean Beads (Vazyme). Libraries were sequenced on an Illumina NovaSeq platform, and 150-bp paired-end reads were generated.

All raw sequence data were quality trimmed using fastp (version 0.19.7) and aligned to the mm10 mouse genome using Bowtie2 (version 2.3.5.1) with options'-local-very-sensitive-local-no-unal-nomixed-no-discordant-phred33-I10-X700'. PCR duplicates were removed using Picard MarkDuplicates (version 2.25.0). Peaks were called using MACS2 (version 2.2.7.1) with options 'q 0.05'. DeepTools2 software (version 3.5.1) was used to create the peaks density plot and heatmap graph. Visualization of peak distribution along genomic regions of interested genes was performed with IGV. Genomic annotation was assigned using ChIPSeeker (version 1.28.3). Promoters were defined as follows: within 3,000 bp around the TSS. Differential expression analysis of two groups was performed using DESeq2 (version 1.30.1).

#### scRNA-seq

300,000 sort-purified ILC3s isolated from the small intestine of *Irf4*^f/f^ and *Irf4*^f/f^ *Rorc*^cre^ were resuspended in BD Pharmingen Stain Buffer (FBS) (554656). The cells were labeled with sample tags using a BD Mouse Immune Single-Cell Multiplexing Kit (633793). Single-cell capture and cDNA synthesis were performed by the BD Rhapsody Single-Cell Analysis System. scRNA-seq libraries were constructed using a BD Rhapsody WTA Amplification Kit (633801) following a standard protocol provided by the manufacturer.

#### scRNA-seq data analysis

The fastq files were processed using the BD Rhapsody Targeted Analysis Pipeline on Seven Bridges. First, the read pair was removed if the mean base quality score was less than 20. Next, the filtered R1 reads were analyzed to identify the cell label sequences and unique molecular identifiers (UMIs). R2 reads were aligned to the mouse genome (mm10) using Bowtie2 (v 2.3.5.1).^53^ Then, valid reads were collapsed into a single raw molecule based on the same cell label, UMI sequence and gene. Recursive substation error correction (RSEC) was applied to correct sequencing and PCR errors of raw UMI counts. The RSEC-adjusted molecule matrices were used for downstream analysis. For quality control, cells with >25% mitochondrial UMI counts or fewer than 200 genes detected were filtered out.

We followed the Scanpy workflow^54^ for downstream analyses. Specifically, we 1) applied the log1pCP10K normalization to the raw counts; 2) selected highly variable genes; 3) regressed out the effects of the total count per cell and the percentage of mitochondrial gene count; 4) calculated the first 50 principal components; 5) applied Harmony^55^ to remove sample-level batch effects; 6) reduced the data dimension through UMAP^56^; 7) clustered the single cells using an unsupervised graph-based clustering algorithm, Leiden^57^; 8) identified cluster-specific marker genes using Student’s t test; and 9) annotated the clusters for their major cell type identities based on the expression patterns of literature-derived marker genes.

Finally, ILC3s were extracted from the integrated atlas by searching for the subset positive for Rorc but negative for Cd3d. Data of ILC3s were then reprocessed from the raw counts and subclustered to identify fine cell states, namely NKp46^+^, CCR6^+^, and CCR6^−^NKp46^-^.

#### Differential abundance testing

To test for ILC3 cell state abundance differences between different mouse groups in an unbiased manner, we performed differential abundance analysis on single cells derived from *Irf4*^f/f^ and *Irf4*^f/f^ *Rorc*^cre^ mice using the milopy package (version 0.0.999).^58^ Specifically, cell neighborhoods were first defined on a *k*-nearest neighbors graph, and differential abundance testing was then performed for each neighborhood using a negative binomial general linear model framework.

#### Pseudo-bulk analysis

To suppress false-positive discoveries in differential expression (DE) analyses using single cells as data points for statistical testing, we employed a pseudo-bulk alternative.^59^ Briefly, for cells of a specific combination of a cell state and a mouse group, we first aggregated reads across biological replicates, transforming a genes-by-cells matrix to a genes-by-replicates matrix using matrix multiplication. Then, we ran DESeq2 (version 1.30.1),^60^ which used a Wald test of the negative binomial model coefficients to compute the statistical significance.

### Quantification and statistical analysis

Statistical parameters, including the exact value of n, the definition of center, dispersion and precision measures and statistical significance, are reported in the figures and figure legends. *p* values less than 0.05 were considered significant. Data from these experiments are presented as mean values ± s.e.m. A two-tailed Student’s t test was performed for comparisons between two groups. All statistical analyses were performed using GraphPad Prism software (version 8, GraphPad Software).
