# Supplementary material for: The transcription factor IRF4 regulates the homeostasis and function of intestinal ILC3s
Source: iScience. 2025 May 31;28(7):112800. doi: 10.1016/j.isci.2025.112800 (PMC12205599; doi:10.1016/j.isci.2025.112800)

iScience, Volume *28*

Supplemental information

The transcription factor IRF4 regulates the homeostasis and function of intestinal ILC3s

Xianzhi Gao, Xin Shen, Qianying Xu, Yan Zeng, Linjia Dong, Shenghui Hong, Huihui Jin, Qianqian Wang, Di Wang, Linrong Lu, and Lie Wang

Figure S1. Phenotype of ILCs in the intestine of IRF4-deficient mice, Related to Figure 1.

(A-B) Protein expression of IRF4 in the indicated ILC subsets isolated from *Irf4*$^{f/f}$ mice. The lineage cocktail included TCRγδ, CD3ε, CD19, CD5, Gr-1, CD11b and Ter119. ILC1: Lin^{-} NKp1.1^{+}NKp46^{+}Eomes^{-} RORγt^{-}; ILC2: Lin^{-}CD127^{+}KLRG1^{+}GATA3^{+}; ILC3: Lin^{-}RORγt^{+}. ILC3 subsets were gated as Lin^{-} RORγt^{+} and then NKp46^{+}, CCR6^{+} or NKp46^{-}CCR6^{-}. (C-D) *C. rodentium* infection model. Flow cytometric analysis (C) and mean fluorescence intensity (MFI) (D) of IRF4 expression in the indicated ILC3 subsets isolated from *Irf4*$^{f/f}$ mice under different infection conditions (n = 5). (E) Protein expression of IRF4 in indicated cell types isolated from the small intestine of *Irf4*$^{f/f}$ and *Irf4*$^{f/f}$ Rorc^{cre} mice. Cells were stimulated for 0.5 h with IL-23 and IL-1β in vitro. (F) Protein expression of IRF4 in ILC3 cells isolated from the small intestine of *Irf4*$^{f/f}$ and *Irf4*$^{f/f}$ Rorc^{cre} mice. (G-H) Flow cytometry of NK, ILC1s, and ILC2s isolated from *Irf4*$^{f/f}$ and *Irf4*$^{f/f}$ *Rorc*^{cre} mice. (I-J) The percentages and total cell numbers of the indicated ILC subsets (n = 5). (K-L) The CD4^{+} ILC3 subset among the CCR6^{+} ILC3s isolated from *Irf4*$^{f/f}$ and *Irf4*$^{f/f}$ *Rorc*^{cre} mice was analyzed (n = 12). Bar graphs are presented as mean ± s.e.m. A two-tailed Student's *t*-test was performed for comparisons. The data are representative of at least three independent experiments (A-L). *P < 0.05, **P < 0.01, ***P < 0.001.

Figure S2. IRF4-deficient ILC3 cells displayed stable proliferation and apoptosis, along with unaltered early differentiation of ILC precursor cells, Related to Figure 1.

(A-B) Proliferation (A) and apoptosis (B) of the indicated ILC3 subsets and isolated from the siLP of *Irf4*$^{f/f}$ and *Irf4*$^{f/f}$ *Rorc*^{cre} mice. (C-D) The percentages of Ki67^{+} (C) and Annexin V^{+} (D) cells in the indicated ILC3 subsets were compared (n = 5). (E-H) Flow cytometric analysis of common lymphoid progenitors (CLPs, Lin^{-}CD127^{+}c-Kit^{int}Sca1^{int}Flt3^{+}); α4β7^{+} lymphoid progenitors (α-LPs, Lin^{-}CD127^{+}c-Kit^{+}α4β7^{+}); common helper-like innate lymphoid progenitors (CHILPs, Lin^{-} CD127^{+}α4β7^{+}CD25^{-}Flt3^{-}) and common ILC precursors (ILCPs, Lin^{-}CD127^{+}α4β7^{+}PLZF^{+}) in bone marrow in *Irf4*$^{f/f}$ and *Irf4*$^{f/f}$ *Rorc*^{cre} mice. The lineage cocktail included TCRγδ, CD3ε, CD19, B220, NK1.1, CD11b, CD11c, Gr-1 and Ter119. The percentages and numbers of CLPs, α-LPs, ChILPs, and ILCPs were compared (n = 4). Bar graphs are presented as mean ± s.e.m. A two-tailed Student's *t*-test was performed for comparisons. The data are representative of at least three independent experiments (A-H).

Figure S3. Validation of NCG adoptive transfer, Related to Figure 5.

(A-B) NCG ($CD45.1^{+}$) mice were adoptively transferred with eighty thousand intestinal ILC3s ($Lin^{-}CD127^{+}CD27^{-}KLRG1^{-}CD45.2^{+}$) sorted from *Irf4*$^{f/f}$ ($CD45.2^{+}$) and *Irf4*$^{f/f}$ *Rorc*^{cre} ($CD45.2^{+}$) mice or PBS as control through the tail vein. All mice were sacrificed for further analysis on day 9. (A) Flow cytometric analysis of donor cells (transferred ILC3s, gated as $CD45.1^{-}Lin^{-}CD45.2^{+}$) isolated from the siLP of NCG control mice (left) or NCG transferred with cells from *Irf4*$^{f/f}$ (middle) and *Irf4*$^{f/f}$ *Rorc*^{cre} (right) mice mice after 9 days post transfer. Initial flow cytometric analysis involved gating on CD45.1-negative populations, which included both adoptively transferred ILC3s and resident non-immune cells within the small intestinal lamina propria of NCG mice. The transferred ILC3 population was then specifically identified through positive selection for CD45.2 expression. (b) The number of donor cells (transferred ILC3s) in the indicated mice was compared. (mean ± SEM; n =4; ***P < 0.001, Student's t test).

Figure S4. The IRF4-deficient mice reveal downregulation of key gene of $NKp46^{+}$ ILC3, Related to Figure 6.

(A) t-SNE plots visualising cell state annotations. (B) t-SNE plots visualising *Irf4* expressions. (C) Stacked bar plot depicting cell state proportions across scRNA-seq samples. (D) Plots display the differences in the three cell state fractions between *Irf4*$^{f/f}$ and *Irf4*$^{f/f}$ *Rorc*^{cre} mice. (E) Violin plots visualising the expression of $CCR6^{+}$ ILC3 signature genes. (F-G) Flow cytometric analysis (F) and MFI (G) of essential factors in *Irf4*$^{f/f}$ and *Irf4*$^{f/f}$ *Rorc*^{cre} mice. (H) Flow cytometric analysis of essential factors. Bar graphs are presented as mean ± s.e.m. A two-tailed Student' s *t*-test was performed for comparisons. The data are representative of at least two independent experiments (F-H). *P < 0.05, **P < 0.01.

Figure S5. MHC-II-related transcriptional signature genes showed a positive correlation with IRF4 expression, Related to Figure 7.

(A) Plot depicting genes positively correlated to IRF4 with an adjusted p-value < 0.01. (b) Activated $CD4^{+}$ T cells were co-cultured with sort-purified ILC3s from the siLP of *Irf4*$^{f/f}$ and *Irf4*$^{f/f}$ *Rorc*^{cre} mice in the presence or absence of Ova peptide or an anti-MHC-II neutralising antibody. FACS analysis of Annexin V^{+} cells. The data are representative of two independent experiments (B).

Figure S6. CUT&Tag of IRF4 binding in intestinal ILC3 subsets, Related to Figure 8.

(A) Pie chart showing the percentages of IRF4-binding at different regions. (B) IGV visualises the indicated gene locus containing ATAC-seq and IRF4-binding peaks in ILC3 subsets. IRF4 CUT & Tag data are from two independent replicates. (C) Motif enrichment analysis within the overlapping peaks of NKp46$^+$ ILC3s, NKp46$^-$CCR6$^-$ ILC3s, and CCR6$^+$ ILC3s in the anti-IRF4 CUT&Tag dataset. The consensus motif, transcription factor name, and p values are shown.

Figures S1

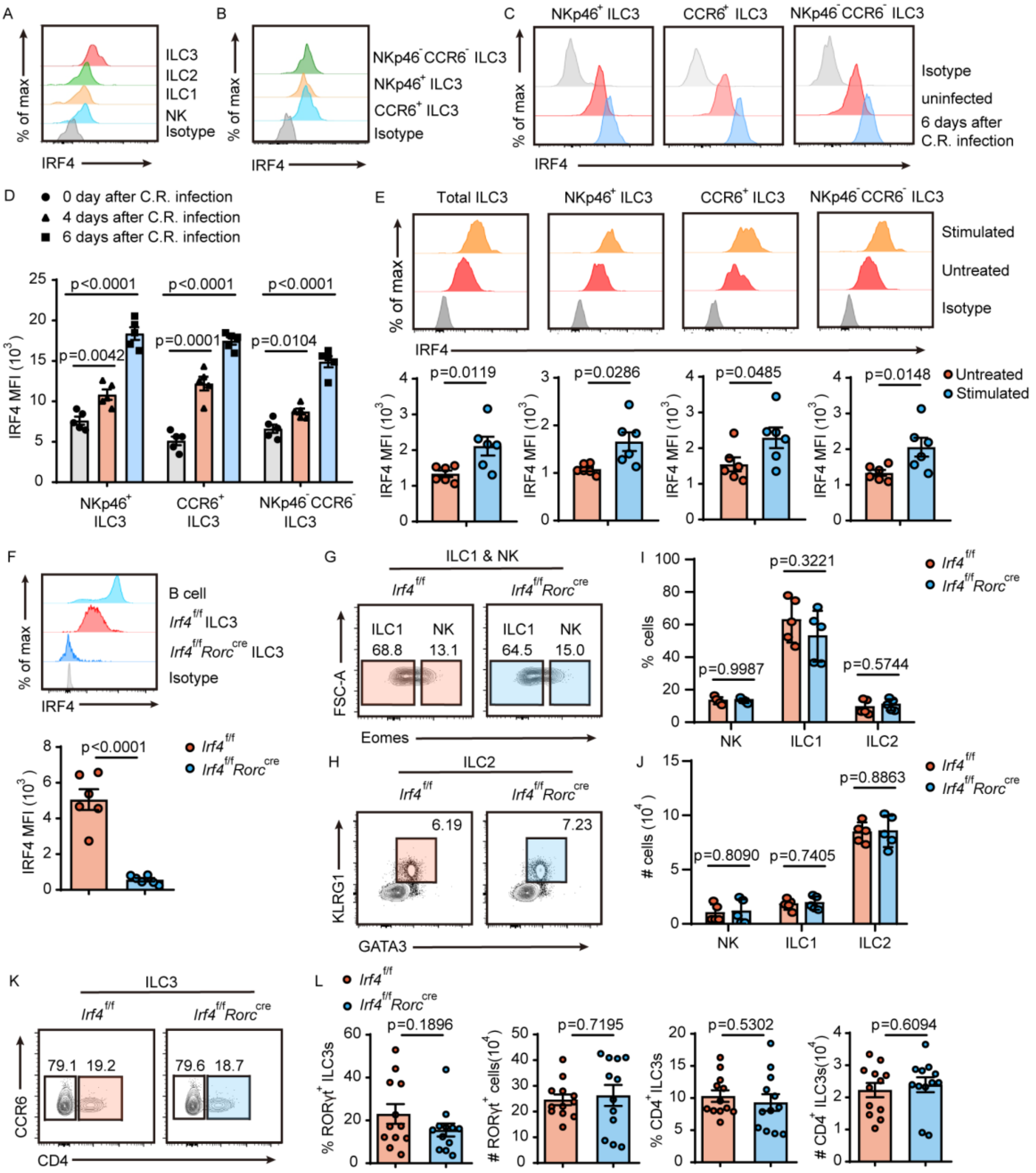

Figures S2

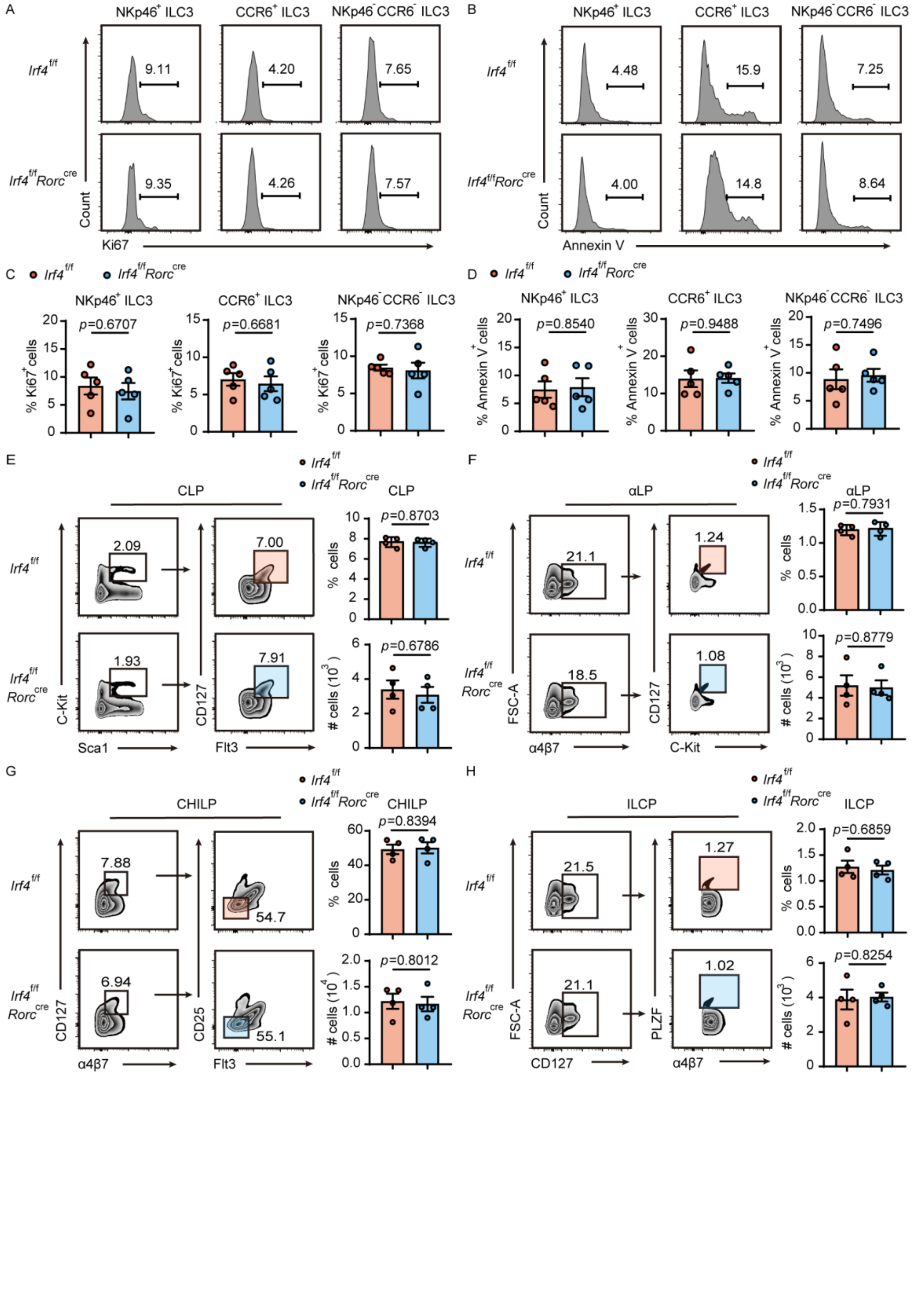
A
NKp46+ ILC3
CCR6+ ILC3
NKp46-CCR6- ILC3
Irf4f/f
Irf4f/fRorccre
Count
Ki67
B
Annexin V
C
D
% Ki67+ cells
% Annexin V+ cells
p=0.6707
p=0.6681
p=0.7368
p=0.8540
p=0.9488
p=0.7496
E
CLP
C-Kit
Sca1
CD127
Flt3
% cells
# cells (10^3)
p=0.8703
p=0.6786
F
αLP
FSC-A
α4β7
C-Kit
p=0.7931
p=0.8779
G
CHILP
CD25
p=0.8394
p=0.8012
# cells (10^4)
H
ILCP
PLZF
p=0.6859
p=0.8254

66

Figures S3

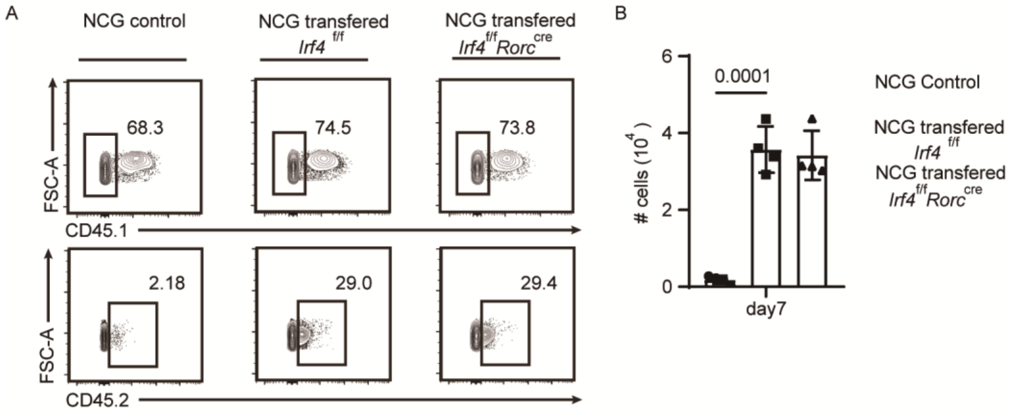

Figures S4

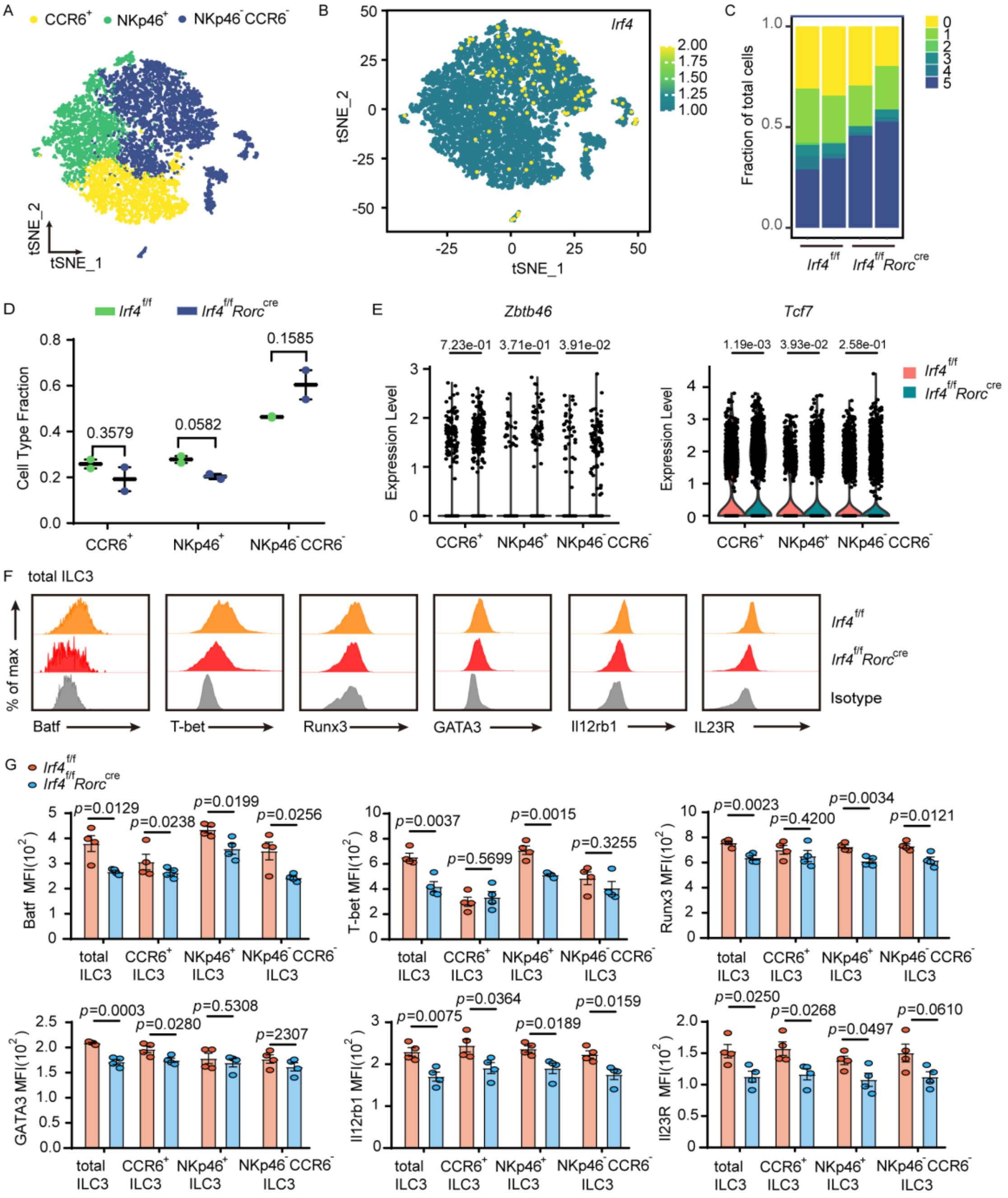
A
CCR6+ NKp46+ NKp46-CCR6-
tSNE_2
tSNE_1
B
Irf4
2.00
1.75
1.50
1.25
1.00
C
Fraction of total cells
0 1 2 3 4 5
Irf4f/f
Irf4f/fRorccre
D
Irf4f/f
Irf4f/fRorccre
Cell Type Fraction
0.3579
0.0582
0.1585
CCR6+
NKp46+
NKp46-CCR6-
E
Zbtb46
Tcf7
7.23e-01
3.71e-01
3.91e-02
1.19e-03
3.93e-02
2.58e-01
Expression Level
F total ILC3
% of max
Batf
T-bet
Runx3
GATA3
Il12rb1
IL23R
Isotype
G
Batf MFI(10^2)
p=0.0129
p=0.0238
p=0.0199
p=0.0256
T-bet MFI(10^2)
p=0.0037
p=0.5699
p=0.0015
p=0.3255
Runx3 MFI(10^2)
p=0.0023
p=0.4200
p=0.0034
p=0.0121
GATA3 MFI(10^2)
p=0.0003
p=0.0280
p=0.5308
p=2307
Il12rb1 MFI(10^2)
p=0.0075
p=0.0364
p=0.0189
p=0.0159
Il23R MFI(10^2)
p=0.0250
p=0.0268
p=0.0497
p=0.0610
total ILC3
CCR6+ ILC3
NKp46+ ILC3
NKp46-CCR6- ILC3

Figures S5

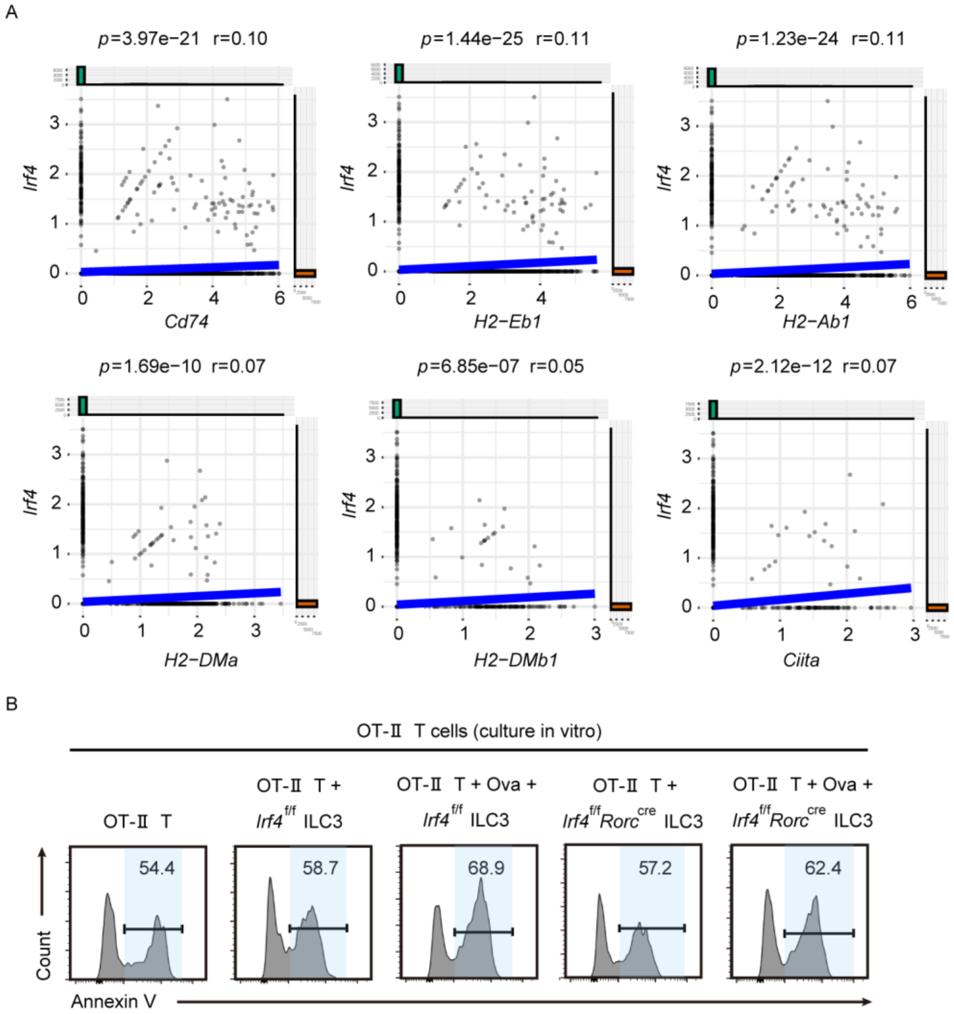

Figures S6

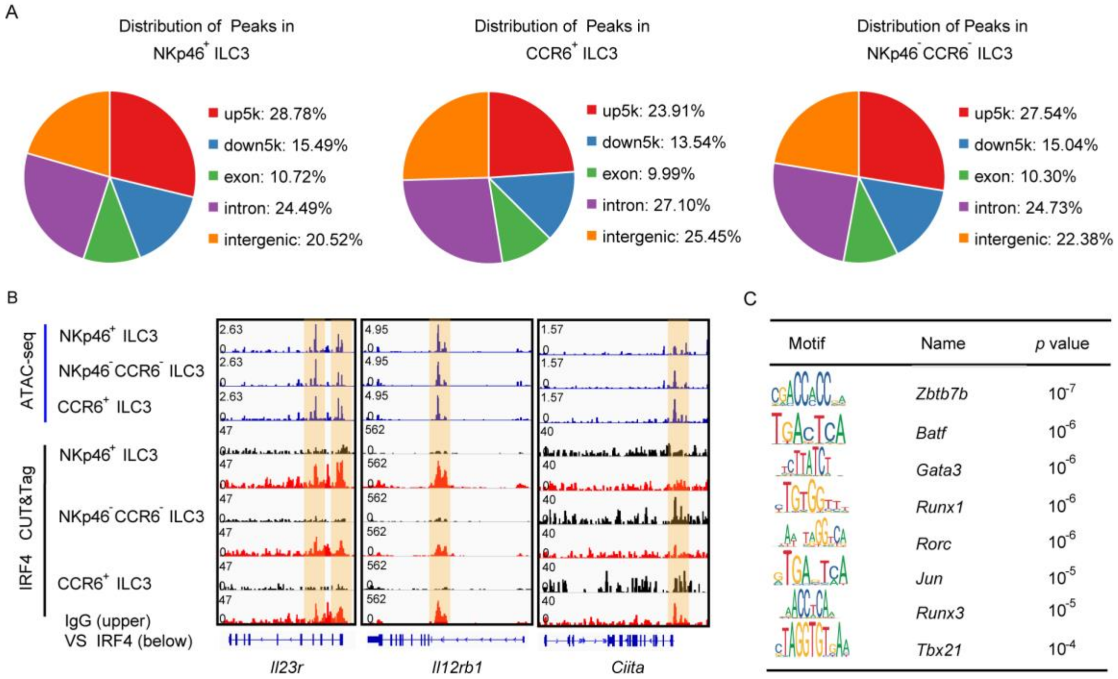

Supplement: Document S1. Figures S1–S6 [file mmc1.pdf]
